# Chemical and structural data of (1,2,3-triazol-4-yl)pyridine-containing coordination compounds

**DOI:** 10.1016/j.dib.2018.08.125

**Published:** 2018-08-30

**Authors:** J. Conradie, M.M. Conradie, K.M. Tawfiq, M.J. Al-Jeboori, C. D'Silva, S.J. Coles, C. Wilson, J.H. Potgieter

**Affiliations:** a Department of Chemistry, University of the Free State, P.O. Box 339, Bloemfontein 9300, South Africa; b Division of Chemistry and Environmental Science, Manchester Metropolitan University, Manchester M1 5GD, UK; c Department of Chemistry, College of Education for Pure Science (Ibn Al-Haitham), University of Baghdad, Baghdad, Iraq; d Manipal University Jaipur, Department of Chemistry, VPO Dehmi Kalan, Jaipur 303007, Rajasthan, India; e EPSRC National Crystallography Service, School of Chemistry, University of Southampton, Southampton SO17 1BJ, England, UK; f School of Chemistry, University of Glasgow, Joseph Black Building, University Avenue, Glasgow G12 8QQ, Scotland, UK; g School of Chemical and Metallurgical Engineering, University of the Witwatersrand, Private Bag X3, Wits 2050, South Africa

## Abstract

The data presented in this paper are related to the research article entitled “Novel dichloro(bis{2-[1-(4-methylphenyl)-1H-1,2,3-triazol-4-yl-κN^3^]pyridine-κN})metal(II) coordination compounds of seven transition metals (Mn, Fe, Co, Ni, Cu, Zn and Cd)” (Conradie et al., 2018) [1]. This paper presents characterization and structural data of the 2-(1-(4-methyl-phenyl)-1H-1,2,3-triazol-1-yl)pyridine ligand (L^2^) (Tawfiq et al., 2014) [2] as well as seven dichloro(bis{2-[1-(4-methylphenyl)-1H-1,2,3-triazol-4-yl-κN^3^]pyridine-κN})metal(II) coordination compounds, [M(L^2^)_2_Cl_2_], all containing the same ligand but coordinated to different metal ions. The data illustrate the shift in IR, UV/VIS, and NMR (for diamagnetic complexes) peaks when L is coordinated to the metals, as well as the influence of the different metals on the peak positions. Solid state structural data is presented for M = Ni and Zn, while density functional theory calculated energies, structures and optimized coordinates are provided for the lowest energy *cis* and *trans* conformations for L^2^ as well as [M(L^2^)_2_Cl_2_] with M = Mn, Fe, Co, Ni, Cu, Zn and Cd.

**Specifications table**

**Table:** 

Subject area	*Chemistry*
More specific subject area	*Coordination compounds*
Type of data	*Table, text file, graph, figure*
How data was acquired	*IR on Thermo-Nicolet FT-IR Spectrometer (AVATAR 320). Mass spectra on WATERS LCT premier mass spectrometer. Magnetic susceptibility with a Gouy magnetic susceptibility balance. X-ray structure on Rigaku SPIDER RAXIS image plate detector and Rigaku AFC12 goniometer equipped with an enhanced sensitivity (HG) Saturn724+ detector mounted at the window of an FR-E+ SuperBright molybdenum rotating anode generator with HF Varimax optics (100 µm focus). NMR on an ECS-400 MHz, JEOL multi nuclear FT spectrometer. UV–vis spectra on a PerkinElmer Lambda 40 UV/Vis spectrometer. Electronic structure calculations using the Gaussian 09 package**[3]*.
Data format	*Raw, calculated, analyzed.*
Experimental factors	–
Experimental features	–
Data source location	*Division of Chemistry and Environmental Science, Manchester Metropolitan University, Manchester, M1 5GD, UK. Department of Chemistry, University of the Free State, Nelson Mandela Street, Bloemfontein, South Africa (DFT). Crystallographic data is held at the NCS University of Southampton. University of Sheffield (MS).*
Data accessibility	*Data is with article.*
Related research article	*J. Conradie, M.M. Conradie, K.M. Tawfiq, M.J. Al-Jeboori, S.J. Coles C. Wilson, J.H. Potgieter, Novel dichloro(bis{2-[1-(4-methylphenyl)-1H-1,2,3-triazol-4-yl-κN*^*3*^*]pyridine-κN})metal(II) coordination compounds of seven transition metals (Mn, Fe, Co, Ni, Cu, Zn and Cd), Polyhedron, 2018, 151 (2018) 243-254.*10.1016/j.poly.2018.03.026.

## Value of the data

• This data would be valuable for the further characterization and structural studies of (1,2,3-triazol-4-yl)pyridine-containing coordination compounds.
• This data provide NMR, IR, UV/VIS and magnetic moment data for (1,2,3-triazol-4-yl)pyridine-containing coordination compounds.
• MS fragmentation data for 2-(1-(4-methyl-phenyl)-1H-1,2,3-triazol-1-yl)pyridine ligand and seven dichloro(bis{2-[1-(4-methylphenyl)-1H-1,2,3-triazol-4-yl- κN^3^]pyridine-κN})metal(II) coordination compounds.
• This data provide solid state structures for two (1,2,3-triazol-4-yl)pyridine-containing coordination compounds.
• This data provide DFT optimized structures and coordinates for the lowest energy *cis* and *trans* isomers of the 2-(1-(4-methyl-phenyl)-1H-1,2,3-triazol-1-yl)pyridine ligand and seven (1,2,3-triazol-4-yl)pyridine-containing coordination compounds.

## 1 Data

### 1.1 Structural data

**Fig. 1:**
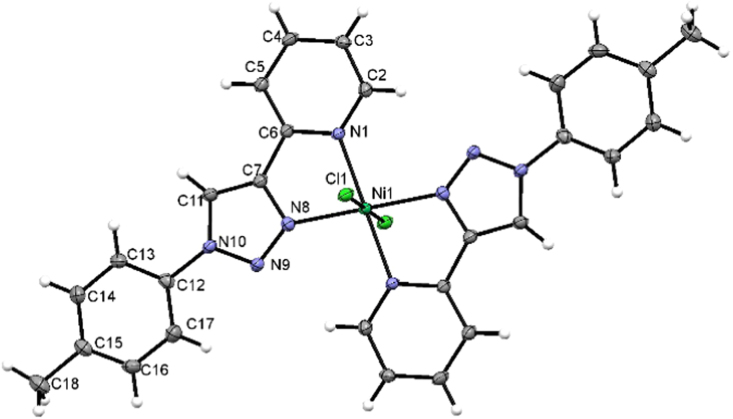
View of [Ni(L^2^)_2_Cl_2_] showing the coordination environment Ni with two 2-(1-(4-methyl-phenyl)-1H-1,2,3-triazol-1-yl)pyridine ligands (L^2^) and two chlorides, as well as the atom labelling scheme used in Table 1.

**Fig. 2:**
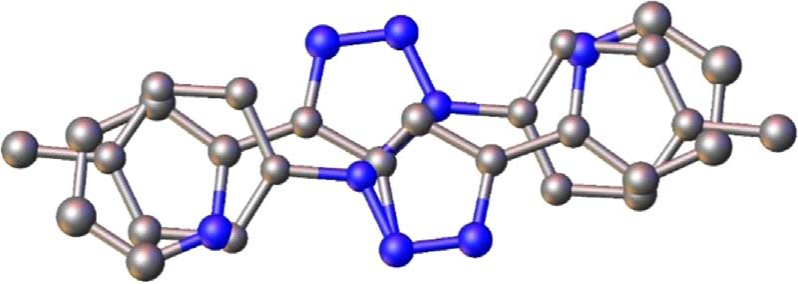
View of the free ligand L = 2-(1-(4-methyl-phenyl)-1H-1,2,3-triazol-1-yl)pyridine in the structure of [Zn(L^2^)_2_Cl_2_].L^2^, disordered over an inversion centre, modelled as 0.5 occupied with isotropic displacement parameters.

The [M(L^2^)_2_Cl_2_] compounds with L^2^ = 2-(1-(4-methyl-phenyl)-1H-1,2,3-triazol-1-yl)pyridine, all have the same chemical formula C_28_H_24_Cl_2_N_8_M with M = Mn, Fe, Co, Ni, Cu, Zn and Cd. The X-ray solid state crystal structure of [Ni(L^2^)_2_Cl_2_] in Fig. 1 shows the coordination environment of the nickel metal ion with two 2-(1-(4-methyl-phenyl)-1H-1,2,3-triazol-1-yl)pyridine ligands L and two chlorides. A list of bond lengths and angles for the ligand L^2^ (that crystallized together with [Zn(L^2^)_2_Cl_2_]), [Zn(L^2^)_2_Cl_2_] and [Ni(L^2^)_2_Cl_2_] are listed in Table 1. The obtained geometrical parameters are in the same range as reported for related complexes [M(L^1^)_2_Cl_2_] with L^1^ = 2-(1-(4-methoxyphenyl)-1H-1,2,3-triazol-1-yl)pyridine and M = Co and Ni [4] and [Ni(L)_2_Br_2_] with L = 1-(cyclohexyl)-4-(2-pyridyl)-1,2,3-triazole [5] (Fig. 2). The obtained geometrical parameters for ligand L^2^ (that crystallized together with [Zn(L^2^)_2_Cl_2_]), are in the same range as reported for ligand L^2^, isolated alone [2].

**Table 1:** Bond lengths [Å] and angles [°] for the ligand and compounds, obtained from solid state structural data [1]. Compound numbering according to Fig. 1.

[Ni(L^2^)_2_Cl_2_]		[Zn(L^2^)_2_Cl_2_]		L^2^ (co-crystallize with [Zn(L^2^)_2_Cl_2_])	
Ni1–N1^i^	2.1015(19)	Zn1–N1	2.144(3)		
Ni1–N1	2.1015(19)	Zn1–N1^i^	2.144(3)		
Ni1–N8^i^	2.0739(19)	Zn1–N8^i^	2.191(4)		
Ni1–N8	2.0739(19)	Zn1–N8	2.191(4)		
Ni1-Cl1	2.4123(6)	Zn1–Cl1^i^	2.4615(14)		
Ni1–Cl1^i^	2.4123(6)	Zn1–Cl1	2.4615(14)		
N1–C2	1.341(3)	N1–C2	1.341(5)	N101–C102	1.270(16)
N1–C6	1.352(3)	N1–C6	1.346(5)	N101–C106	1.373(16)
N8–N9	1.316(3)	N8–N9	1.316(5)	N108–N109	1.315(11)
N8–C7	1.357(3)	N8–C7	1.363(5)	N108–C107	1.379(12)
N9–N10	1.352(3)	N9–N10	1.364(5)	N109–N110	1.378(12)
N10–C11	1.353(3)	N10–C11	1.352(5)	N110–C111	1.347(14)
N10–C12	1.428(3)	N10–C12	1.434(5)	N110–C112	1.433(13)
C2–C3	1.383(3)	C2–C3	1.385(6)	C102–C103	1.388(16)
C2–H2	0.9300	C2–H2	0.930	C102–H102	0.930
C3–C4	1.385(3)	C3–C4	1.381(6)	C103–C104	1.366(16)
C3–H3	0.9300	C3–H3	0.930	C103–H103	0.930
C4–C5	1.381(3)	C4–C5	1.378(6)	C104–C105	1.33(2)
C4–H4	0.9300	C4–H4	0.930	C104–H104	0.930
C5-C6	1.388(3)	C5–C6	1.403(6)	C105–C106	1.39(2)
C5–H5	0.9300	C5–H5	0.930	C105–H105	0.930
C6–C7	1.460(3)	C6–C7	1.458(6)	C106–C107	1.451(14)
C7–C11	1.363(3)	C7–C11	1.369(6)	C107–C111	1.405(16)
C11–H11	0.9300	C11–H11	0.930	C111–H111	0.930
C12–C13	1.383(3)	C12–C13	1.376(6)	C112–C113	1.387(13)
C12–C17	1.384(3)	C12–C17	1.383(6)	C112–C117	1.338(16)
C13–C14	1.386(3)	C13–C14	1.397(6)	C113–C114	1.39(2)
C13–H13	0.9300	C13–H13	0.930	C113–H113	0.930
C14–C15	1.385(4)	C14–C15	1.385(7)	C114–C115	1.40(2)
C14–H14	0.9300	C14–H14	0.930	C114–H114	0.930
C15–C16	1.389(4)	C15–C16	1.388(7)	C115–C116	1.368(15)
C15–C18	1.508(3)	C15–C18	1.526(6)	C115–C118	1.502(14)
C16–C17	1.379(4)	C16–C17	1.390(7)	C116–C117	1.462(17)
C16–H16	0.9300	C16–H16	0.930	C116–H116	0.930
C17–H17	0.9300	C17–H17	0.930	C117–H117	0.930
C18–H18A	0.9600	C18–H18A	0.960	C118–H11A	0.960
C18–H18B	0.9600	C18–H18B	0.960	C118–H11B	0.960
C18–H18C	0.9600	C18–H18C	0.960	C118–H11C	0.960
N8i–Ni1–N8	180.0	N1–Zn1–N1i	180.0		
N8–Ni1–N1i	100.41(8)	N1–Zn1–N8i	77.78(13)		
N8–Ni1–N1	79.59(8)	N1i–Zn1–N8	102.22(13)		
N1i–Ni1–N1	180.0	N8i–Zn1–N8	180.0		
N8^i^–Ni1–Cl1	90.20(6)	N1–Zn1–Cl1^i^	90.83(9)		
N8–Ni1–Cl1	89.80(6)	N1^i^–Zn1–Cl1^i^	89.17(9)		
N1^i^–Ni1–Cl1	89.38(6)	N8^i^–Zn1–Cl1^i^	88.93(10)		
N1–Ni1–Cl1	90.62(6)	N8–Zn1–Cl1^i^	91.07(10)		
N8^i^–Ni1–Cl1^i^	89.80(6)	N1–Zn1–Cl1	89.17(9)		
N8–Ni1–Cl1^i^	90.20(6)	N1^i^–Zn1–Cl1	90.83(9)		
N1^i^–Ni1–Cl1^i^	90.62(6)	N8^i^–Zn1–Cl1	91.07(10)		
N1–Ni1-Cl1^i^	89.38(6)	N8–Zn1–Cl1	88.93(10)		
Cl1–Ni1–Cl1^i^	180.00(2)	Cl1^i^–Zn1–Cl1	180.0		
C2–N1–Ni1	127.46(16)	C2–N1–Zn1	125.5(3)		
C6–N1–Ni1	114.55(15)	C6–N1–Zn1	115.4(3)		
N9–N8–Ni1	137.61(16)	N9–N8–Zn1	138.7(3)		
C7–N8–Ni1	112.60(15)	C7–N8–Zn1	111.1(3)		
C2–C3–C4	119.2(2)	C4–C3–C2	119.1(4)	C104–C103–C102	118.1(13)
C2–C3–H3	120.4	C4–C3–H3	120.4	C102–C103-H103	120.9
C2–N1–C6	117.9(2)	C2–N1–C6	119.0(4)	C102–N101-C106	119.4(12)
C3–C2–H2	118.8	C3–C2–H2	119.1	C103–C102-H102	118.5
C3–C4–H4	120.4	C3–C4–H4	120.0	C103–C104-H104	119.9
C4–C3–H3	120.4	C2–C3–H3	120.4	C104–C103-H103	120.9
C4–C5–C6	118.4(2)	C4–C5–C6	117.7(4)	C104–C105-C106	119.2(16)
C4–C5–H5	120.8	C4–C5–H5	121.1	C104–C105–H105	120.4
C5–C4–C3	119.2(2)	C5–C4–C3	120.1(4)	C105–C104–C103	120.2(14)
C5–C4–H4	120.4	C5–C4–H4	120.0	C105–C104–H104	119.9
C5–C6–C7	123.0(2)	C5–C6–C7	122.0(4)	C105–C106–C107	123.8(11)
C6–C5–H5	120.8	C6–C5–H5	121.1	C106–C105–H105	120.4
C7–C11–H11	127.7	C7–C11–H11	127.3	C107–C111–H111	127.4
C11–C7–C6	132.6(2)	C11–C7–C6	132.4(4)	C111–C107–C106	130.7(10)
C11–N10–C12	127.9(2)	C11–N10–C12	129.0(4)	C111–N110–C112	129.9(7)
C12–C13–C14	118.7(2)	C12–C13–C14	119.7(5)	C112–C113–C114	118.0(11)
C12–C13–H13	120.6	C12–C13–H13	120.2	C112–C113–H113	121.0
C12–C17–H17	120.3	C16–C17–H17	120.3	C112–C117–H117	120.9
C13–C12–C17	120.6(2)	C13–C12–C17	120.3(4)	C117–C112–C113	123.2(10)
C13–C12–N10	119.2(2)	C13–C12–N10	120.0(4)	C113–C112–N110	119.0(9)
C13–C14–H14	119	C13–C14–H14	119.5	C113–C114–H114	119.2
C14–C13–H13	120.6	C14–C13–H13	120.2	C114–C113–H113	121.0
C14–C15–C16	117.8(2)	C14–C15–C16	118.3(5)	C116–C115–C114	119.1(13)
C14–C15–C18	121.4(2)	C14–C15–C18	121.4(5)	C114–C115–C118	119.6(12)
C15–C14–C13	121.9(2)	C15–C14–C13	121.0(5)	C113–C114–C115	121.7(15)
C15–C14–H14	119	C15–C14–H14	119.5	C115–C114–H114	119.2
C15–C16–H16	119.2	C17–C16–H16	119.3	C115–C116–H116	120.1
C16–C15–C18	120.8(2)	C16–C15–C18	120.4(5)	C116–C115–C118	121.2(10)
C16–C17–C12	119.4(2)	C12–C17–C16	119.5(5)	C112–C117–C116	118.3(12)
C16–C17–H17	120.3	C12–C17–H17	120.3	C116–C117–H117	120.9
C17–C12–N10	120.2(2)	C17–C12–N10	119.7(4)	C117–C112–N110	117.8(10)
C17–C16–C15	121.5(2)	C15–C16–C17	121.3(5)	C115–C116–C117	119.7(11)
C17–C16–H16	119.2	C15–C16–H16	119.3	C117–C116–H116	120.1
N10–C11–C7	104.5(2)	N10–C11–C7	105.3(4)	N110–C111–C107	105.2(8)
N10–C11–H11	127.7	N10–C11–H11	127.3	N110–C111–H111	127.4
N1–C2–C3	122.4(2)	N1–C2–C3	121.8(4)	N101–C102–C103	123.0(13)
N1–C2–H2	118.8	N1–C2–H2	119.1	N101–C102–H102	118.5
N1–C6–C5	122.9(2)	N1–C6–C5	122.2(4)	N101–C106–C105	120.0(12)
N1–C6–C7	114.2(2)	N1–C6–C7	115.8(4)	N101–C106–C107	116.2(10)
N8–C7–C11	108.4(2)	N8–C7–C11	107.7(4)	N108–C107–C111	107.8(9)
N8–C7–C6	119.1(2)	N8–C7–C6	119.8(4)	N108–C107–C106	121.5(8)
N8–N9–N10	106.02(18)	N8–N9–N10	106.1(3)	N108–N109–N110	107.9(8)
N9–N10–C11	111.3(2)	N9–N10–C11	110.7(3)	C111-N110-N109	110.2(7)
N9–N10–C12	120.81(19)	N9–N10–C12	120.2(3)	N109–N110–C112	119.9(9)
N9–N8–C7	109.79(19)	N9–N8–C7	110.2(4)	N109–N108–C107	108.9(8)
C15–C18–H18A	109.5	C15–C18–H18A	109.5		
C15–C18–H18B	109.5	C15–C18–H18B	109.5		
C15–C18–H18C	109.5	C15–C18–H18C	109.5		
H18A–C18–H18B	109.5	H18A–C18–H18B	109.5		
H18A–C18–H18C	109.5	H18A–C18–H18C	109.5		
H18B–C18–H18C	109.5	H18B–C18–H18C	109.5		
Symmetry transformations used to generate equivalent atoms	(i) -x+1,-y+1,-z+1	(i) -x,-y+1,-z+1		(i) -x,-y+1,-z+1

### 1.2 Spectroscopic data

**Fig. 3:**
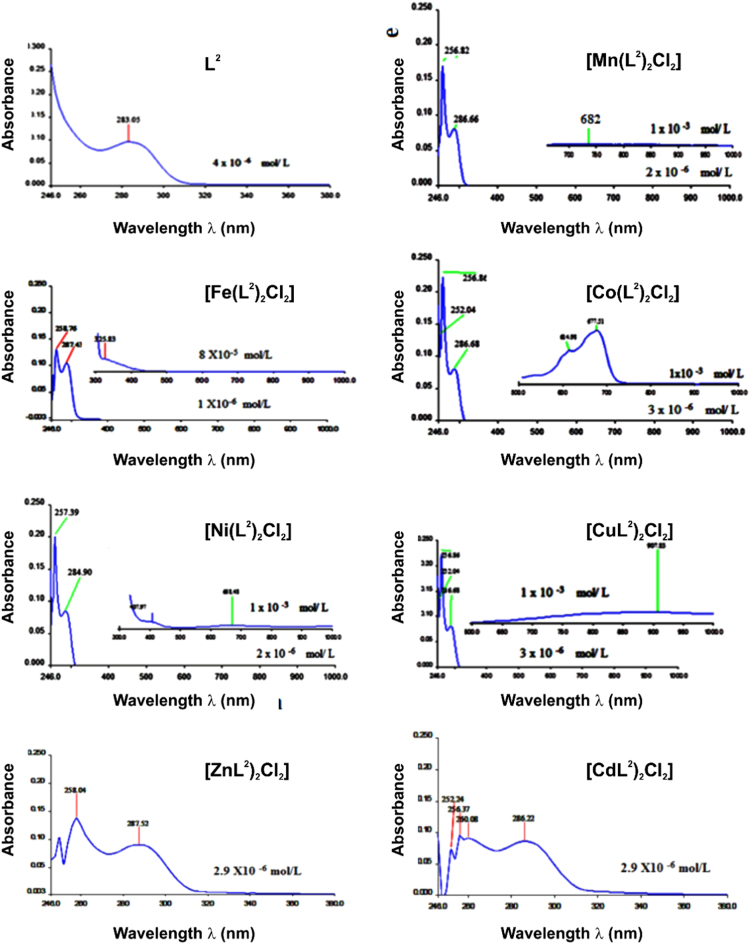
UV–vis spectra of L^2^ and [M(L^2^)_2_Cl_2_] in DMSO solutions.

**Fig. 4:**
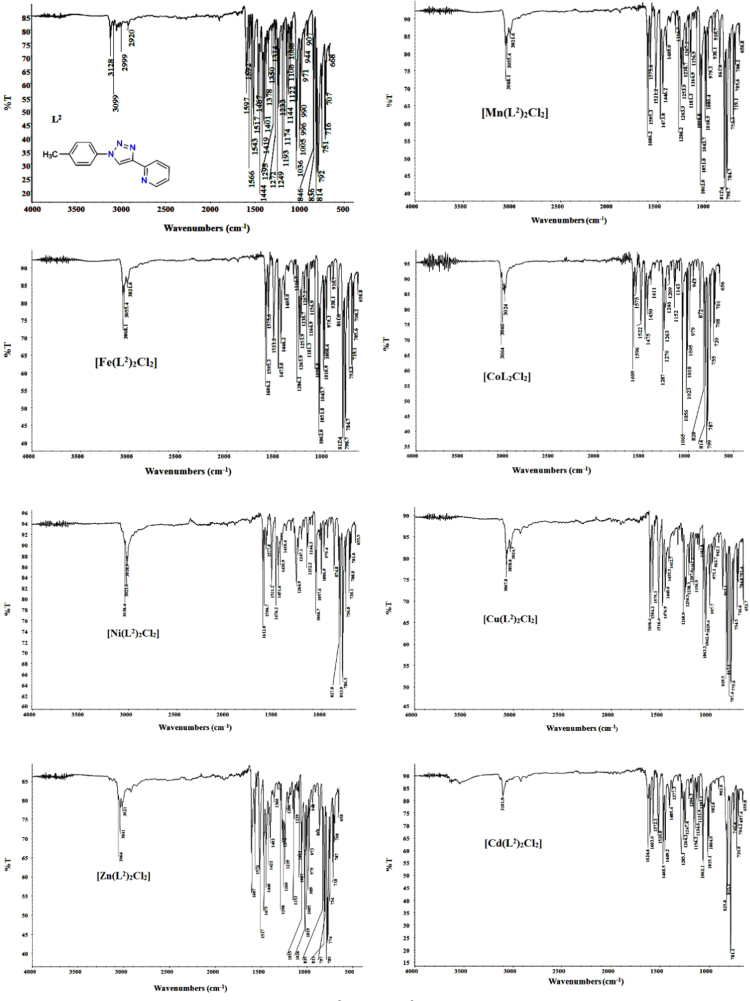
FT-IR absorption spectra of L^2^ and [M(L^2^)_2_Cl_2_] compounds.

**Fig. 5:**
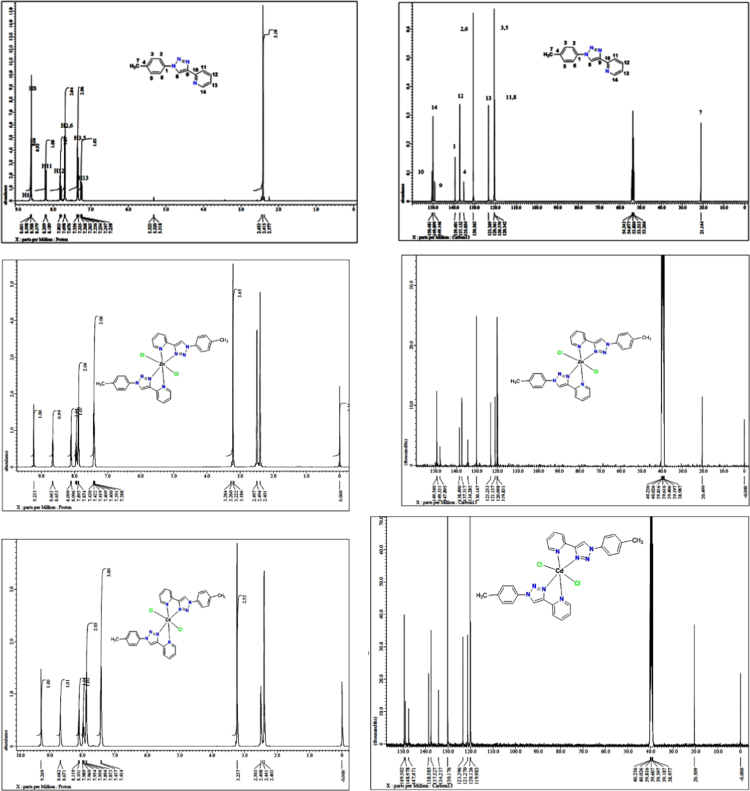
^1^H and ^13^C NMR spectrum of L^2^ in CD_2_Cl_2_, [Zn(L^2^)_2_Cl_2_] and [Cd(L^2^)_2_Cl_2_] in DMSO-d_6_.

The UV/vis spectra of L^2^ and the [M(L^2^)_2_Cl_2_] compounds are shown in Fig. 3 and characteristic data is summarized in Table 2. The IR spectra of L^2^ and the [M(L^2^)_2_Cl_2_] compounds are shown in Fig. 4. Selected characteristic IR bands of L^2^ and the [M(L^2^)_2_Cl_2_] compounds are listed and compared in reference [1]. The ionization data of the TOFMS-ES (+) mass spectra of L^2^ and the [M(L^2^)_2_Cl_2_] compounds given are summarized in Table 3. The TOFMS-ES (+) mass spectra are provided in the Supplementary material. The ^1^H and ^13^C NMR spectra of L^2^ and the diamagnetic [M(L^2^)_2_Cl_2_] compounds (M = Zn or Cd) are shown in Fig. 5, while data to determine the spin state (amount of unpaired d-electrons) for the paramagnetic [M(L^2^)_2_Cl_2_] compounds (M = Mn, Fe, Co, Ni and Cu) are summarized in Table 4. More NMR spectra are provided in the Supplementary material.

**Table 2:** UV–vis spectral data and assignments of L^2^ and [M(L^2^)_2_Cl_2_] in DMSO solutions.

**Compound**	**Band Position λ**_**max nm**_	**Wave number (cm**^**-1**^**)**	**Extinction coefficient ε**_**max**_**(**_**dm**_^**3**^_**mol**_^**-1**^_**cm**_^**-1**^**)**	**Assignment**
L^2^	258, 287	38759, 34843	19740, 17200 (4 × 10^-5^ M)	Intra-ligand π→π*, n→π*
[Mn(L^2^)_2_Cl_2_]	280, 284	35714, 35211	3165, 3124(1 × 10^-4^ M)	Intra-ligand π→π*, n→π*
	682	14662	13	^6^A_1_g^(S)^ →^4^T_1_g^(4G)^
[Fe(L^2^)_2_Cl_2_]	284	35211	29513 (1.2 × 10^-4^ M)	Intra-ligand π→π*, n→π*
	326	30674	4947	CT
[Co(L^2^)_2_Cl_2_]	280, 286, 298	35714, 34965, 33557	3672, 3347,3240 (1 × 10^-3^ M)	Intra-ligand π→π*, n→π*
	615	1620	56	^4^T_1_g ^(F)^ → ^4^T_1_g^( P)^
	677	14970	89	^4^T_1_g ^(F)^ → ^4^A_2_g ^(F)^
[Ni(L^2^)_2_Cl_2_]	278, 282, 300	35971, 35460, 33333	3602, 3653, 3656 (1 × 10^3^ M)	Intra-ligand π→π*, n→π*
	408	24509	20	^3^A_2_g ^(F)^ → ^3^T_1_g ^(p)^
	668	14970	8	^3^A_2_g ^(F)^ → ^3^T_1_g ^(F)^
[Cu L^2^)_2_Cl_2_]	279, 284	35842,35211	3507, 3603 (1 × 10^-3^ M)	Intra-ligand π→π*, n→π*
	310	32258	3696	–
	908	11013	85	^2^B_1_g → ^2^B_2_ g
[Zn(L^2^)_2_Cl_2_]	260, 287	38461, 35843	3220, 3067 (4 × 10^-5^ M)	Intra-ligand π→π*, n→π*
[Cd(L^2^)_2_Cl_2_]	259, 287	38759, 34843	28005, 25695 (4 × 10^-5^ M)	Intra-ligand π→π*, n→π*

**Table 3:** Fragmentation data of positive electrospray ionization of L^2^ and the metals coordination compounds with ligand L^2^.

**L**^**2**^**or coordination compound**	**Formula**	**MW**	**Fragmentation,*****m/z*****(%)**
L^2^	C_14_H_12_N_4_	236.3	209 [M-N_2_]^+^ 22%, 237 [M+H]^+^ 100%, 259 [M+Na]^+^ 7%, 495 [2 M+Na]^+^ 18% (consistend with literature [6])
[Mn(L^2^)_2_Cl_2_]	C_28_H_24_Cl_2_MnN_8_	598.4	562.1 [M-Cl]^+^ 90%, calculated for [(C_28_H_24_N_8_MnCl)]^+^, 237.1 [L^2^]^+^ 70%, calculated for [(C_14_H_12_N_4_)]^+^, 209.1 [L^2^- N_2_] ^+^ calculated for [(C_14_H_14_N_2_)]^+^ 100%
[Fe(L^2^)_2_Cl_2_]	C_28_H_24_Cl_2_FeN_8_	599.3	563.1 [M-Cl]^+^ 90%, calculated for [(C_28_H_24_N_8_FeCl)]^+^, 237.1 [L^2^]^+^ 70%, calculated for [(C_14_H_12_N_4_)]^+^, 209.1 [L^2^-N_2_] ^+^ calculated for [(C_14_H_14_N_2_)]^+^ 100%
[Co(L^2^)_2_Cl_2_]	C_28_H_24_Cl_2_CoN_8_	602.4	566.1 [M-Cl]^+^ 40%, calculated for [(C_28_H_24_N_8_CoCl)]^+^, 531 [M-Cl_2_]^+^ 5%, calculated for (C_28_H_24_CoN_8_)
[Ni(L^2^)_2_Cl_2_]	C_28_H_24_Cl_2_NiN_8_	602.1	565.1 [M-Cl]^+^ 40%, calculated for [(C_28_H_24_N_8_NiCl)]^+^, 265 [M-Cl_2_-L^2^+N_2_]^+^ 50%, calculated for [(C_14_H_10_N_2_Ni)]^+^, 209.1 [L^2^-N_2_]^+^ (10%), calculated for [(C_14_H_12_N_2_)]^+^.
[Cu(L^2^)_2_Cl_2_]	C_28_H_24_Cl_2_CuN_8_	607.0	594.1 [M-Cl]^+^45%, calculated for [(C_28_H_24_N_8_CuCl)]^+^, 535.14[Cu(L^2^)_2_]^+^30%, calculated for [C_28_H_24_CuN_8_]^+^ 30%, 358 [CuL^2^]^+^, calculated for [C_14_H_12_N_4_ Cu CH_3_COO^-^]^+^ 100%, 237 [L^2^]^+^ calculated for [(C_14_H_12_N_4_)]^+^ 40%, 209 [L^2^-N_2_]^+^ 15%, calculated for [(C_14_H_12_N_2_)]^+^.
[Zn(L^2^)_2_Cl_2_]	C_28_H_24_Cl_2_ZnN_8_	608.8	571.2 [M-Cl]^+^ (80%), calculated for [(C_28_H_24_N_8_ZnCl)]^+^, 33[M-Cl-(L^2^)]^+^ 5%, 237.1 [L^2^]^+^, calculated for [(C_14_H_12_N_4_Zn)]^+^ 30%, 209.1 [L^2^- N_2_]^+^ calculated for [(C_14_H_12_N_2_)] ^+^ 90%.
[Cd(L^2^)_2_Cl_2_]	C_28_H_24_Cl_2_CdN_8_	655.9	621.2[M-Cl]^+^ (100%), calculated for [(C_28_H_24_N_8_CdCl)]^+^, 237.1 [L^2^] ^+^ (20%), calculated for [(C_14_H_12_N_4_)]^+^, 209.1 [L^2^- N_2_]^+^ calculated for [(C_14_H_12_N_2_)]^+^ 50%

**Table 4:** Data for determination of the spin state of paramagnetic [M(L^2^)_2_Cl_2_] complexes, **µ**_**eff**_ = effective magnetic moment.

**Compound**	**amount of d electrons**	**µ**_**eff**_**measured (B.M)**	***µ***_**eff**_ = $\sqrt{S(S+1)}$**calculated (B.M)**	***S***
[Mn(L^2^)_2_Cl_2_]	5	5.62	5.92	5/2
[Fe(L^2^)_2_Cl_2_]	6	5.26	4.90	2
[Co(L^2^)_2_Cl_2_]	7	3.98	3.87	3/2
[Ni(L^2^)_2_Cl_2_]	8	3.00	2.83	1
[Cu L^2^)_2_Cl_2_]	9	1.70	1.73	1/2

### 1.3 DFT data

**Fig. 6:**
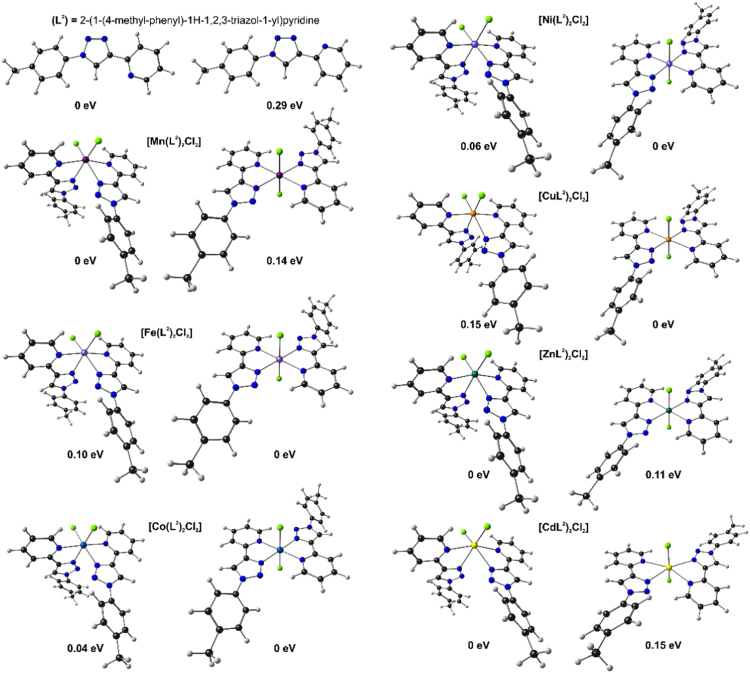
Density functional theory calculated optimized geometries of the lowest energy *cis* and *trans* isomers of L^2^ and the [M(L^2^)_2_Cl_2_]. The relative energies of the isomers, ΔE in eV, is also shown; the energy of the lowest energy isomer is indicated as 0 eV.

Both L^2^ and the [M(L^2^)_2_Cl_2_] complexes may have different stereoisomers. The density functional theory calculated lowest energy *cis* and *trans* isomers, as well as the relative energies of the isomers, are shown in Fig. 6. The data associated with the geometry of the optimized geometries (Cartesian coordinates) of the compounds shown are provided in the Supplementary material.

## 2 Experimental design, materials, and methods

Density functional theory (DFT) calculations were performed in the gas phase on the neutral compounds, using the B3LYP functional and the triple-ζ basis set 6–311 G(d,p) on all atoms except for Cd where the Stuttgart/Dresden (SDD) pseudopotential was used to describe the metal electronic core, while the metal valence electrons were described using the def2-TZVPP basis set [7]. The Gaussian 09 package [3] were used to optimize the compounds. The multiplicity used for L^2^ and the [M(L^2^)_2_Cl_2_] compounds is singlet (L^2^, [Zn(L^2^)_2_Cl_2_] and [Cd(L^2^)_2_Cl_2_]), doublet ([Cu(L^2^)_2_Cl_2_]), triplet ([Ni(L^2^)_2_Cl_2_]), quartet ([Co(L^2^)_2_Cl_2_]), quintet ([Fe(L^2^)_2_Cl_2_]) and sextet ([Mn(L^2^)_2_Cl_2_]).
