# Supplementary material for: Chemical and structural data of (1,2,3-triazol-4-yl)pyridine-containing coordination compounds
Source: Data Brief. 2018 Aug 30;20:1397–408. doi: 10.1016/j.dib.2018.08.125 (PMC6148730; doi:10.1016/j.dib.2018.08.125)
Supplement: Supplementary file 1 — Supplementary material [file mmc1.docx]

# Declarations of interest

none
