# Supplementary material for: Chemical and structural data of (1,2,3-triazol-4-yl)pyridine-containing coordination compounds
Source: Data Brief. 2018 Aug 30;20:1397–408. doi: 10.1016/j.dib.2018.08.125 (PMC6148730; doi:10.1016/j.dib.2018.08.125)
Supplement: Supplementary file 2 — Supplementary Table. [file mmc2.docx]

**Data Article - Supplementary material**

**Title**: Chemical and structural data of (1,2,3-triazol-4-yl)pyridine-containing coordination compounds.

**Authors**: J. Conradie^1*^, M.M. Conradie^1^, K.M. Tawfiq^2^, M.J. Al-Jeboori^3^, C. D'Silva^4^, S.J. Coles^5^ C. Wilson^6^ and J.H. Potgieter^2,7*^

**Affiliations**:

1. Department of Chemistry, University of the Free State, P.O. Box 339, Bloemfontein, 9300, South Africa

2. Division of Chemistry and Environmental Science, Manchester Metropolitan University, Manchester, M1 5GD, UK

3. Department of Chemistry, College of Education for Pure Science (Ibn Al-Haitham), University of Baghdad, Baghdad, Iraq

4. Manipal University Jaipur, Department of Chemistry, VPO Dehmi Kalan, Jaipur, Rajasthan, PIN 303007, India.

5. EPSRC National Crystallography Service, School of Chemistry, University of Southampton, Southampton, SO17 1BJ, England

6. School of Chemistry, University of Glasgow, Joseph Black Building. University Avenue, Glasgow, G12 8QQ, Scotland

7. School of Chemical and Metallurgical Engineering, University of the Witwatersrand, Private Bag X3, Wits, 2050, South Africa

**Contact email**: conradj@ufs.ac.za

Table of Contents

[**Data Article - Supplementary material** 1](#_Toc509328657)

[Mass spectra for L^2^. 3](#_Toc509328658)

[Mass spectra for [M(L^2^)_2_Cl_2_]. 4](#_Toc509328659)

[Dept NMR, 2D ^1^H-^13^C HMQC and 2D ^1^H-^1^HCOSY correlation spectral for the ligand L^2^. 11](#_Toc509328660)

[Dept NMR, 2D ^1^H-^13^C HMQC and 2D ^1^H-^1^HCOSY correlation spectral for [Zn(L^2^)_2_Cl_2_]. 13](#_Toc509328661)

[Dept NMR, 2D ^1^H-^13^C HMQC and 2D ^1^H-^1^HCOSY correlation spectral for [Cd(L^2^)_2_Cl_2_]. 15](#_Toc509328662)

[Optimized Cartesian coordinates (Å). 17](#_Toc509328663)

[1. L^2^ S = 0 *cis* isomer 17](#_Toc509328664)

[2. L^2^ S = 0 *trans* isomer 18](#_Toc509328665)

[3. [Mn(L^2^)_2_Cl_2_] S = 5/2 *cct* isomer (R = CH_3_) 18](#_Toc509328666)

[4. [Mn(L^2^)_2_Cl_2_] S = 5/2 *ttt* isomer (R = CH_3_) 20](#_Toc509328667)

[5. [Fe(L^2^)_2_Cl_2_] S = 2 *cct* isomer (R = CH_3_) 21](#_Toc509328668)

[6. [Fe(L^2^)_2_Cl_2_] S = 2 *ttt* isomer (R = CH_3_) 22](#_Toc509328669)

[7. [Co(L^2^)_2_Cl_2_] S = 3/2 *cct* isomer (R = CH_3_) 24](#_Toc509328670)

[8. [Co(L^2^)_2_Cl_2_] S = 3/2 *ttt* isomer (R = CH_3_) 25](#_Toc509328671)

[9. [Ni(L^2^)_2_Cl_2_] S = 1 *cct* isomer (R = CH_3_) 26](#_Toc509328672)

[10. [Ni(L^2^)_2_Cl_2_] S = 1 *ttt* isomer (R = CH_3_) 28](#_Toc509328673)

[11. [Cu(L^2^)_2_Cl_2_] S = 1/2 *cct* isomer (R = CH_3_) 29](#_Toc509328674)

[12. [Cu(L^2^)_2_Cl_2_] S = 1/2 *ttt* isomer (R = CH_3_) 30](#_Toc509328675)

[13. [Zn(L^2^)_2_Cl_2_] S = 0 *cct* isomer (R = CH_3_) 32](#_Toc509328676)

[14. [Zn(L^2^)_2_Cl_2_] S = 0 *ttt* isomer (R = CH_3_) 33](#_Toc509328677)

[15. [Cd(L^2^)_2_Cl_2_] S = 0 *cct* isomer (R = CH_3_) 34](#_Toc509328678)

[16. [Cd(L^2^)_2_Cl_2_] S = 0 *ttt* isomer (R = CH_3_) 36](#_Toc509328679)

[17. [Zn(L^2^)_2_Cl_2_] di-molecular model S = 0 *ttt* isomer (R = CH_3_) 37](#_Toc509328680)

# Mass spectra for L^2^.


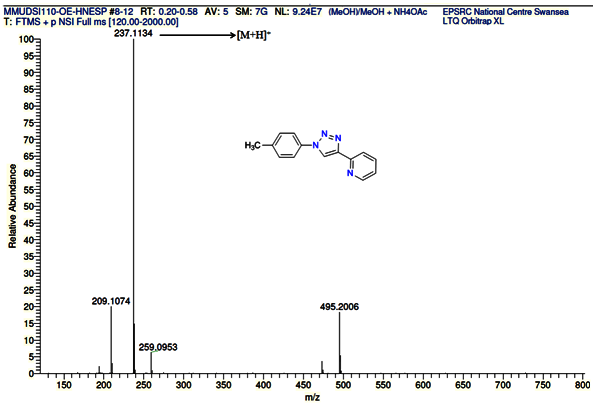


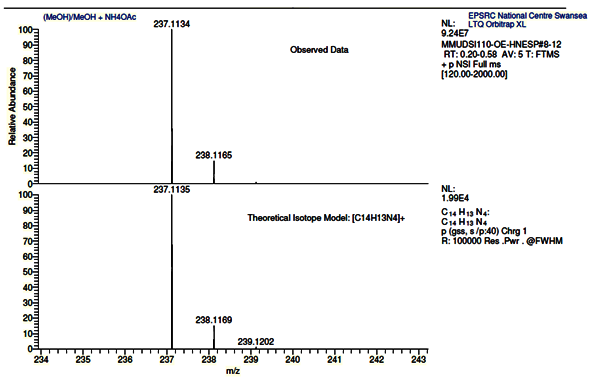


**Figure S 17. Top: Mass spectral of 2-(1- p-tolyl)-1H-1,2,3-triazol-4-yl)-pyridine (L^2^). Middle and bottom: The calculated accurate mass spectrum of (L^2^)**

# Mass spectra for [M(L^2^)_2_Cl_2_].


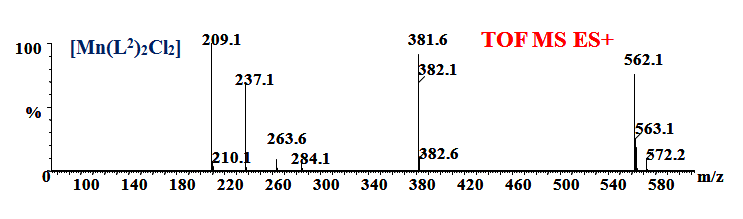


**
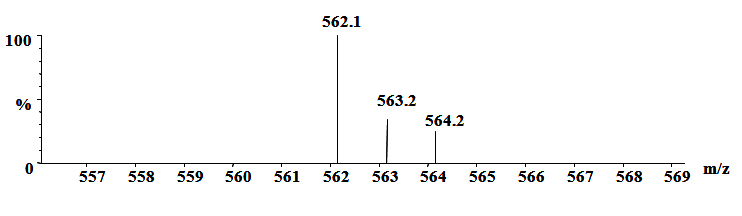

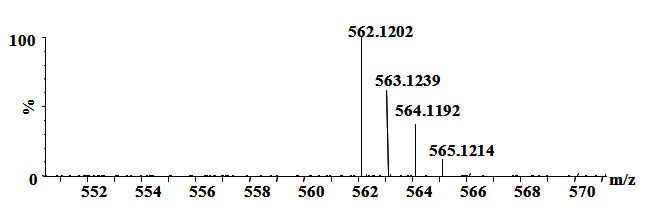
**

**Figure S 18. Top: TOFMS-ES (+) mass spectrum of coordination compound [Mn(L^2^)_2_Cl_2_]. Middle and bottom: The calculated mass spectrum of [Mn(L^2^)_2_Cl_2_].**

**
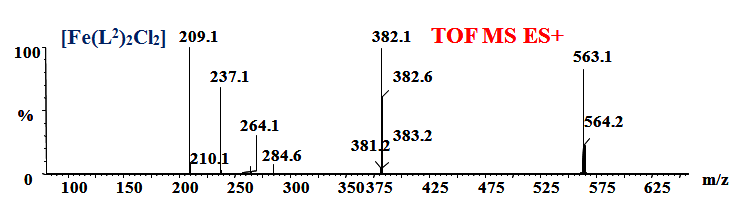
**

**
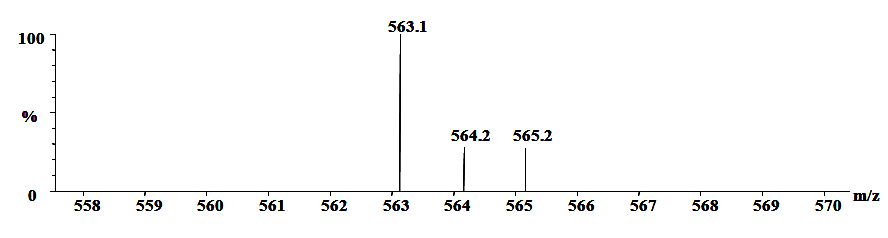
**

**
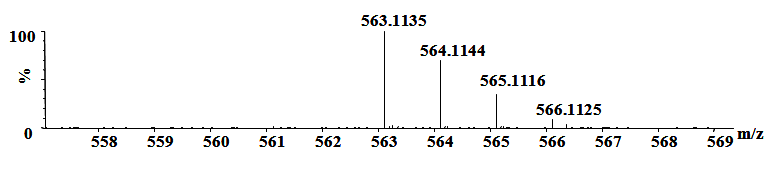
**

**Figure S 19. Top: TOFMS-ES (+) mass spectrum of coordination compound [Fe(L^2^)_2_Cl_2_]. Bottom: The calculated mass spectrum of [Fe(L^2^)_2_Cl_2_].**

**
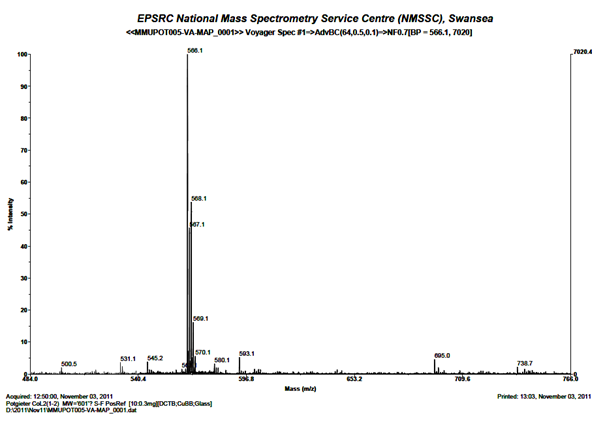

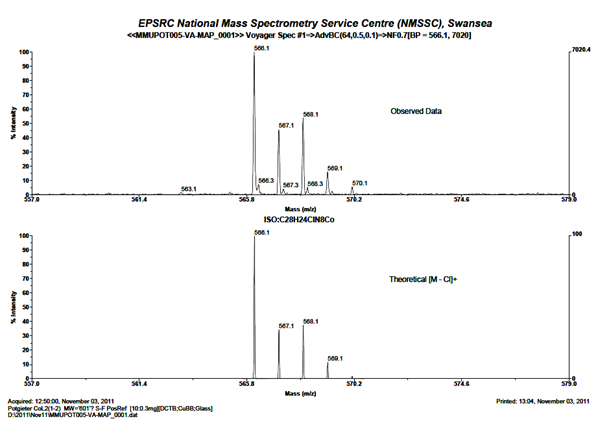
Figure S 20. Top: TOFMS-ES (+) mass spectrum of coordination compound [Co(L^2^)_2_Cl_2_]. Middle and bottom: The calculated mass spectrum of [Co(L^2^)_2_Cl_2_].**

**
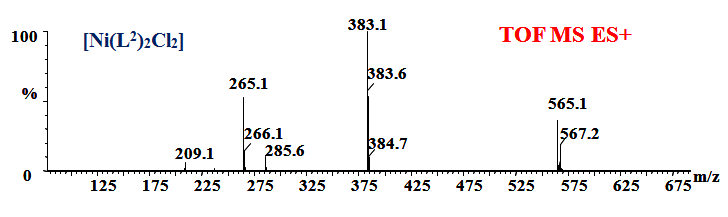
**

**
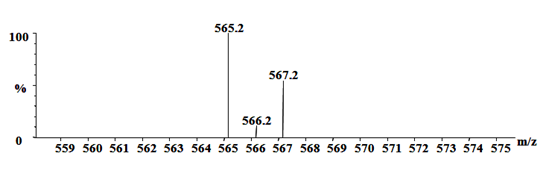
**

**
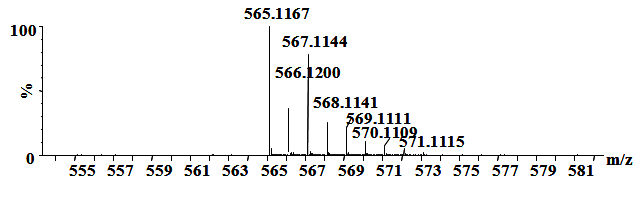
**

**Figure S 21. Top: TOFMS-ES (+) mass spectrum of coordination compound [Ni(L^2^)_2_Cl_2_]. Middle and bottom: The calculated mass spectrum of [Ni(L^2^)_2_Cl_2_].**

**
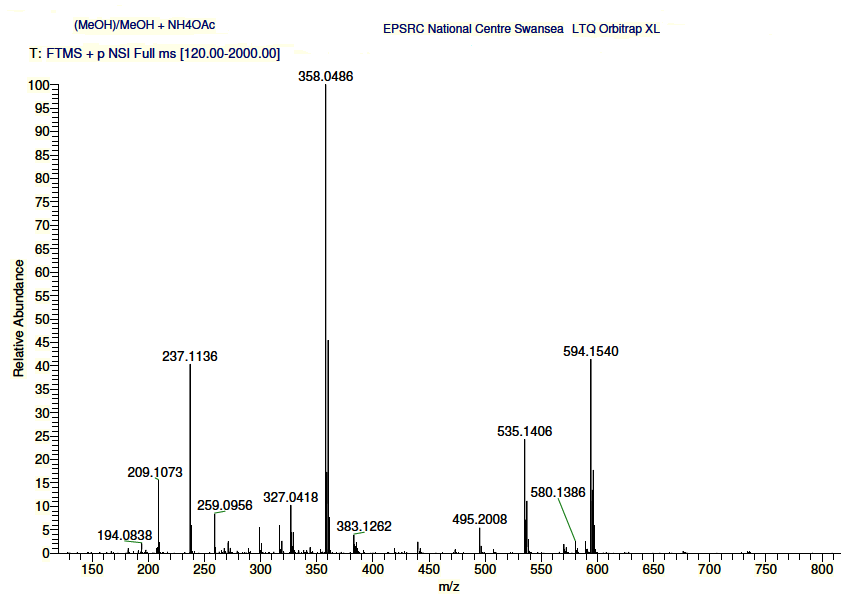
**

**
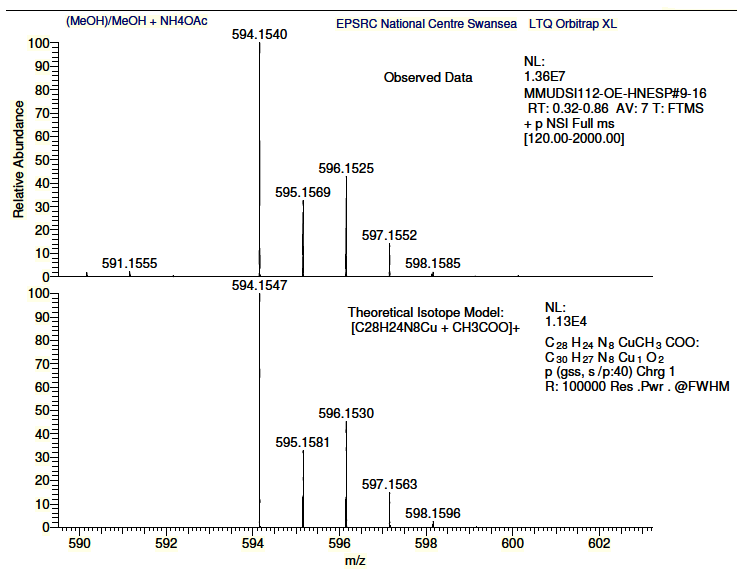
**

**Figure S 22. Top: TOFMS-ES (+) mass spectrum of coordination compound [Cu(L^2^)_2_Cl_2_]. Middle and bottom: The calculated mass spectrum of [Cu(L^2^)_2_Cl_2_].**

**
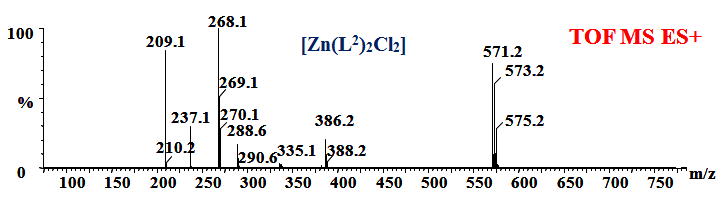
**

**
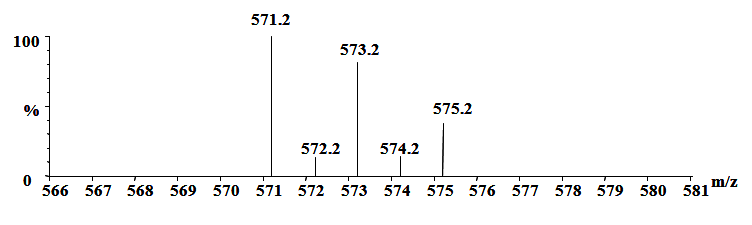
**

**
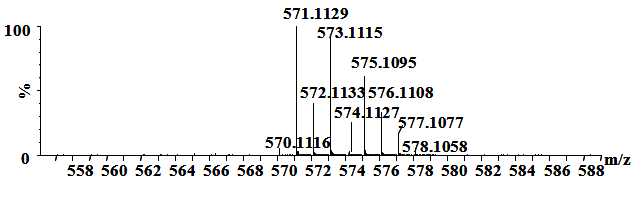
**

**Figure S 23. Top: TOFMS-ES (+) mass spectrum of coordination compound [Zn(L^2^)_2_Cl_2_]. Middle and bottom: The calculated mass spectrum of [Zn(L^2^)_2_Cl_2_].**

**
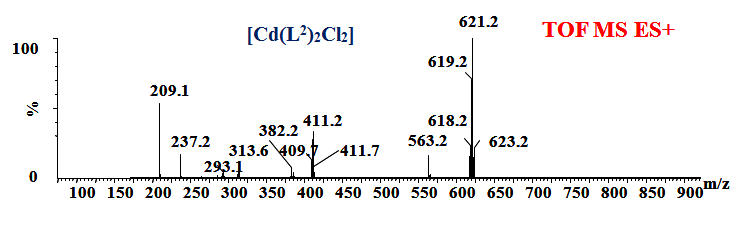
**

**
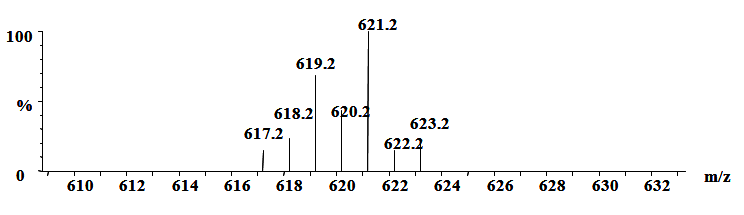
**

**
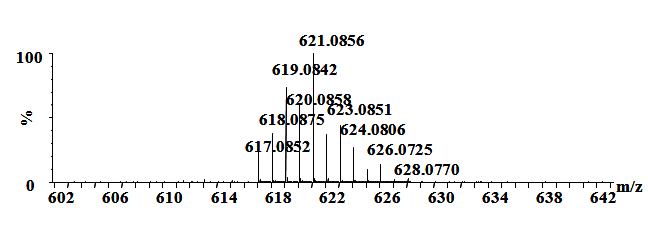
**

**Figure S 24. Top: TOFMS-ES (+) mass spectrum of coordination compound [Cd(L^2^)_2_Cl_2_]. Middle and bottom: The calculated mass spectrum of [Cd(L^2^)_2_Cl_2_].**

# Dept NMR, 2D ^1^H-^13^C HMQC and 2D ^1^H-^1^HCOSY correlation spectral for the ligand L^2^.

**
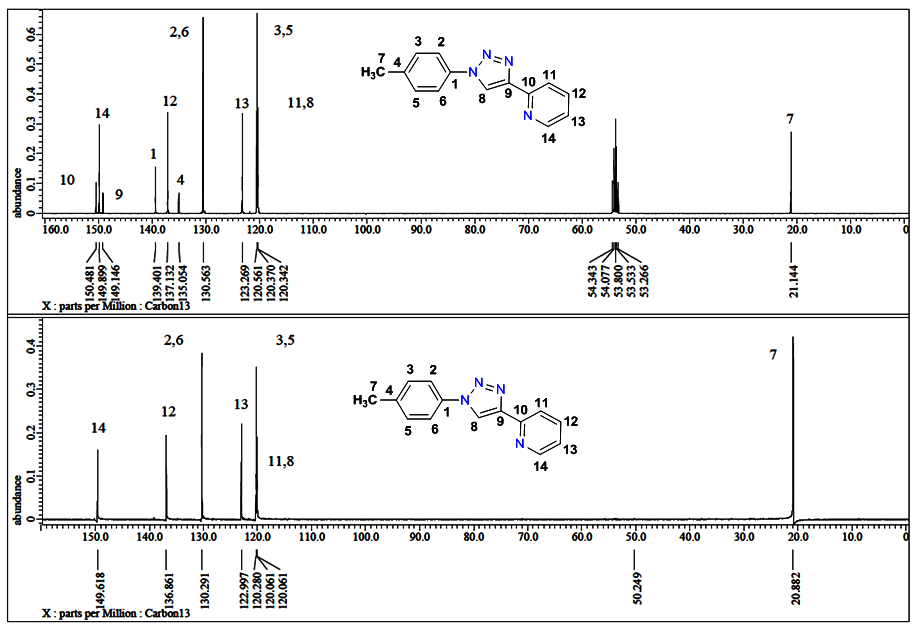
**

Figure S 27. **The ^13^C NMR and DEPT NMR spectrum of L^2^ in CD_2_Cl_2_.**

**_
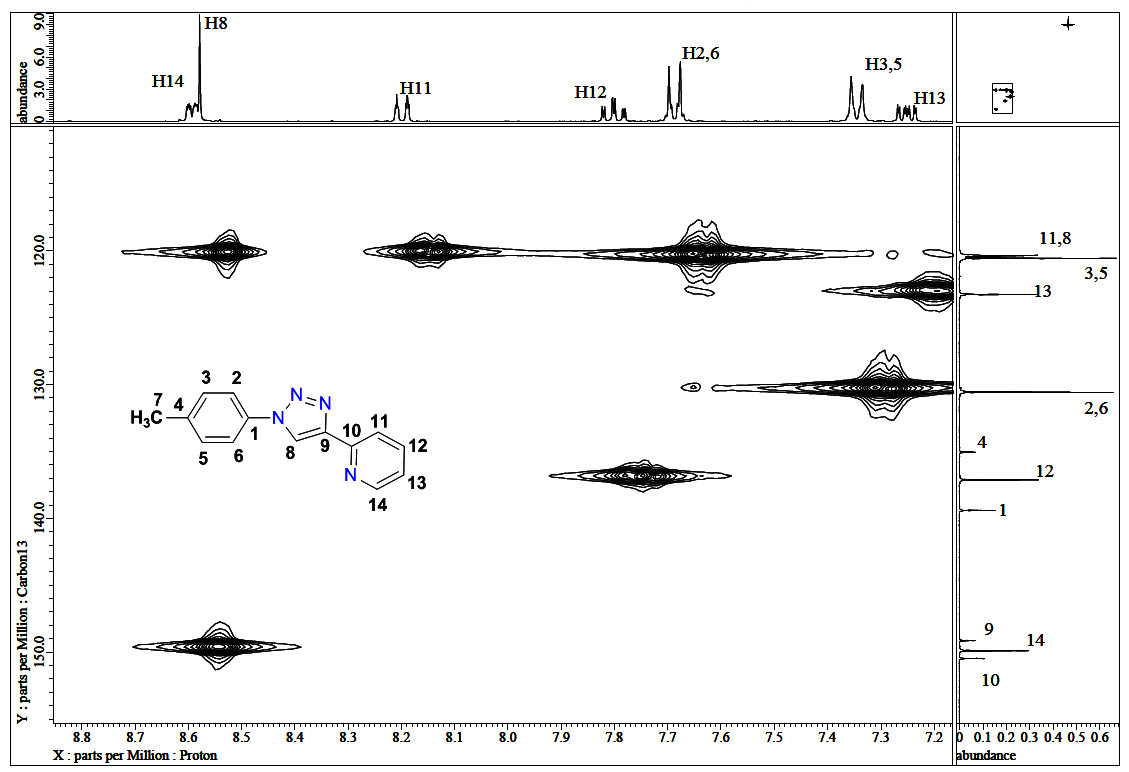
_**

Figure S 28. **The 2D ^1^H-^13^C HMQC correlation spectrum of L^2^ in CD_2_Cl_2_.**


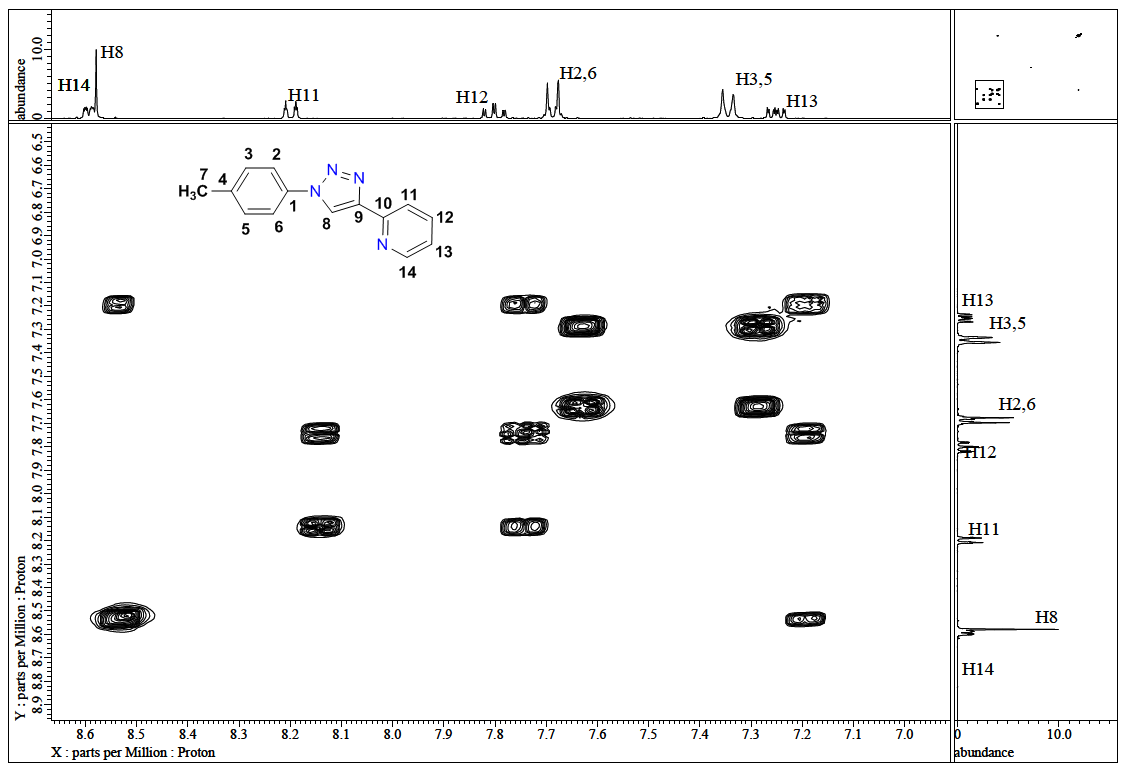


Figure S 29. **The 2D ^1^H-^1^HCOSY** **correlation spectrum of L^2^ in CD_2_Cl_2_.**

# Dept NMR, 2D ^1^H-^13^C HMQC and 2D ^1^H-^1^HCOSY correlation spectral for [Zn(L^2^)_2_Cl_2_].

**
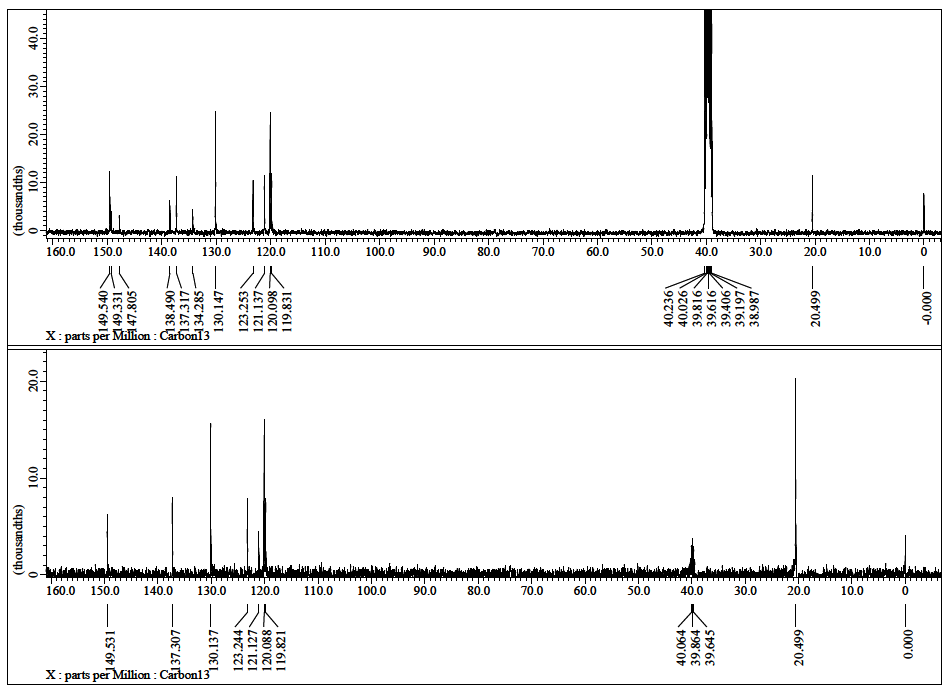
**

Figure S 32. **The ^13^C NMR and dept NMR spectrum of [Zn(L^2^)_2_Cl_2_] in DMSO-d6**

**
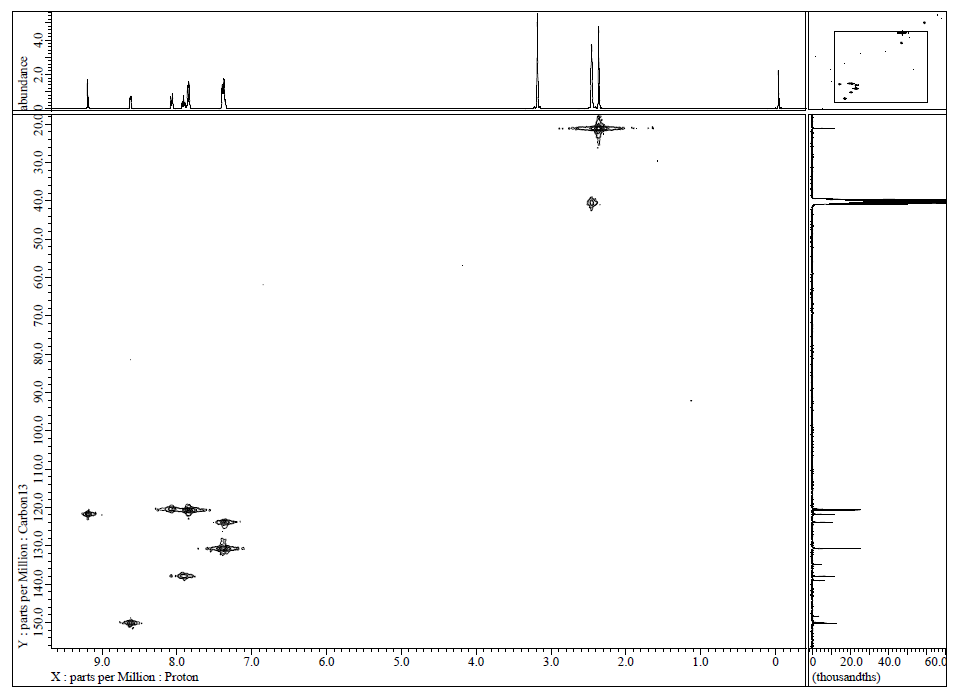
**

Figure S 33. **The 2D ^1^H-^13^C HMQC correlation spectrum of [Zn(L^2^)_2_Cl_2_] in DMSOd_6_**

**^
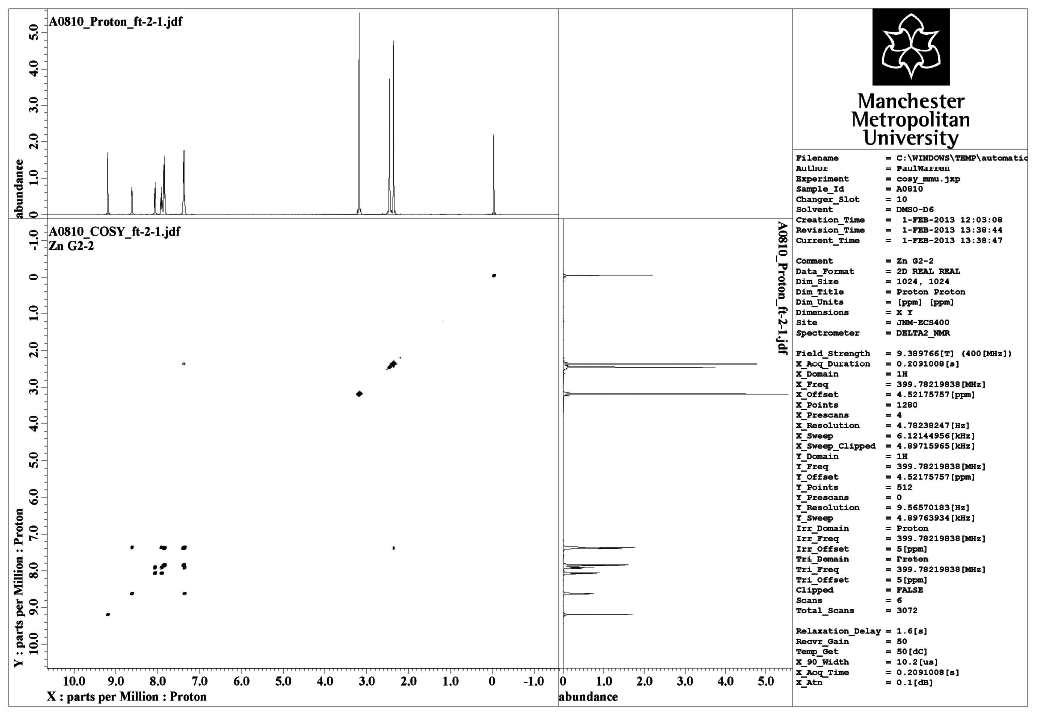
^**

Figure S 34. **The 2D ^1^H-^1^H COSY** **correlation spectrum of [Zn(L^2^)_2_Cl_2_] in DMSOd_6_**

# Dept NMR, 2D ^1^H-^13^C HMQC and 2D ^1^H-^1^HCOSY correlation spectral for [Cd(L^2^)_2_Cl_2_].

**
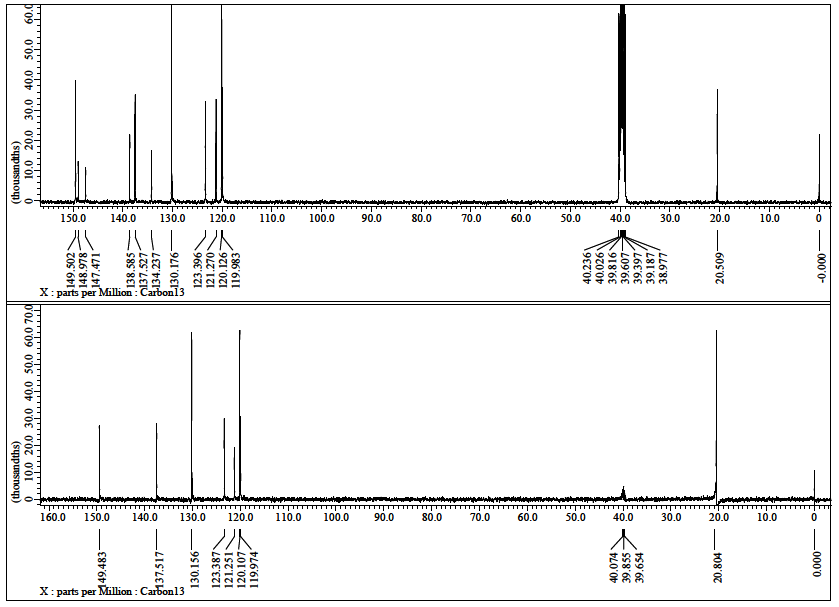
**

Figure S 37. **The ^13^C NMR and dept NMR spectrum of [Cd(L^2^)_2_Cl_2_] in DMSO-d6**

**
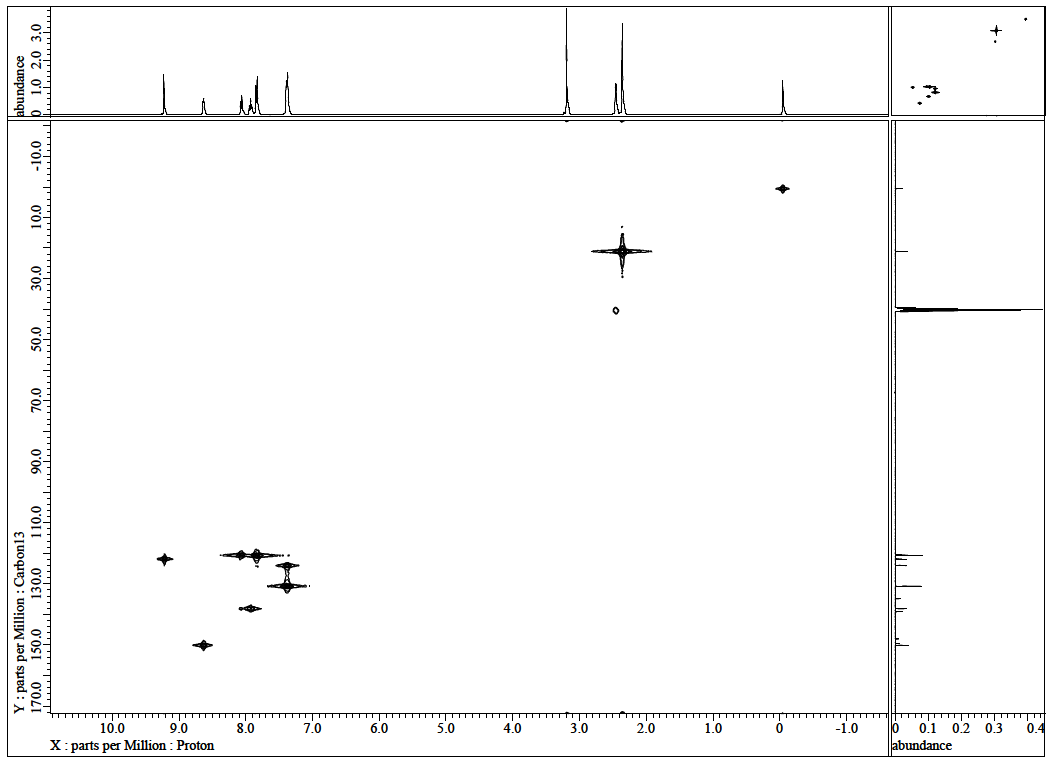
**

Figure S 38. **The 2D ^1^H-^13^C HMQC correlation spectrum of [Cd(L^2^)_2_Cl_2_] in DMSOd_6_**

**_
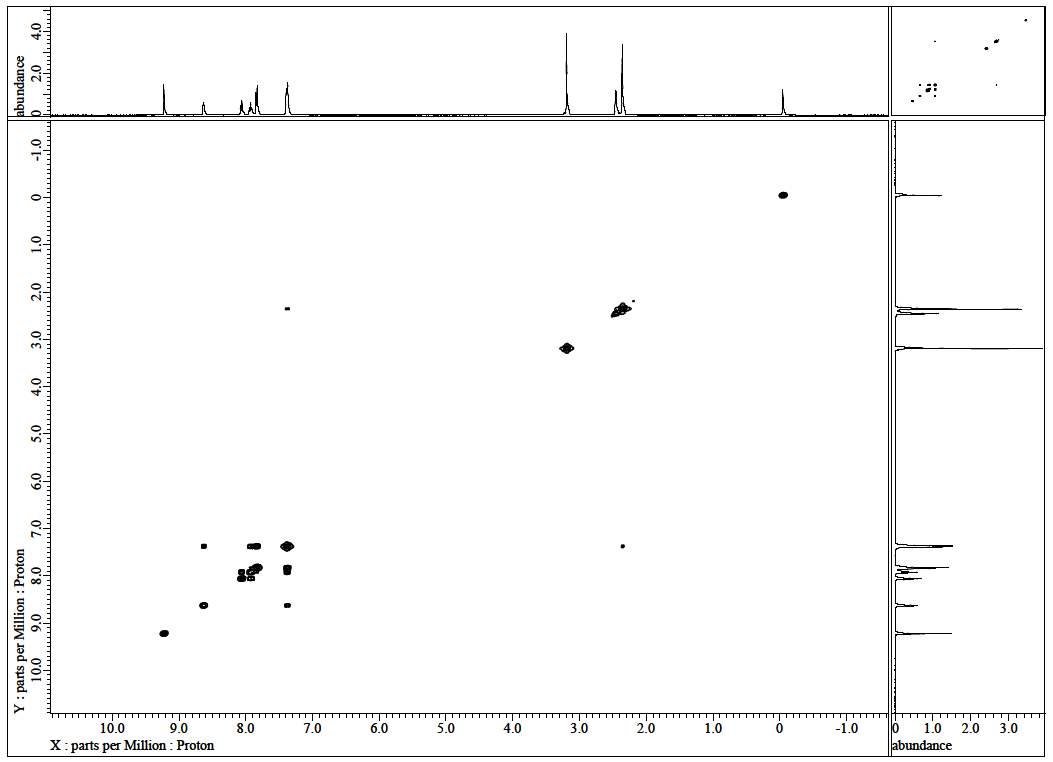
_**

Figure S 39. **The 2D ^1^H-^1^HCOSY** **correlation spectrum of [Cd(L^2^)_2_Cl_2_] in DMSOd_6_**

# Optimized Cartesian coordinates (Å).

Density functional theory (DFT) calculations were performed with the B3LYP functional as implemented in the Gaussian 09 package [^[[1]](#endnote-1)^] using the triple-ζ basis set 6-311G(d,p), except for Cd where the Stuttgart/Dresden (SDD) pseudopotential was used to describe the metal electronic core, while the metal valence electrons were described using the def2-TZVPP basis set [^[[2]](#endnote-2)^].

Figure S 42. The various dichloro(bis{2-[1-(4-R-phenyl)-1H-1,2,3-triazol-4-yl-kN3]pyridine-kN})metal(II) coordination compounds, M = Mn (1), Fe (2), Co (3), Ni (4), Cu (5), Zn (6) and Cd (7). R = H for the unsubstituted ligand, L^1^, and R = CH_3_ for the CH_3_ substituted ligand, L^2^.

# L^2^ S = 0 *cis* isomer

symmetry c1

C -2.011231000 -0.147967000 -0.077659000

C -2.492432000 1.138831000 -0.308317000

H -1.813778000 1.937510000 -0.581953000

C -3.858778000 1.389487000 -0.216303000

H -4.223027000 2.394658000 -0.399415000

C -4.766068000 0.372025000 0.087022000

C -4.258376000 -0.916313000 0.296920000

H -4.940461000 -1.727852000 0.528175000

C -2.897969000 -1.182813000 0.224854000

H -2.510178000 -2.178782000 0.389226000

C -6.245667000 0.647152000 0.197343000

H -6.574091000 0.620189000 1.241671000

H -6.830304000 -0.100265000 -0.345618000

H -6.499028000 1.630217000 -0.204001000

C 0.436086000 0.402552000 0.072450000

H 0.314925000 1.420583000 0.396926000

C 1.551245000 -0.383846000 -0.133892000

C 2.972652000 -0.022924000 -0.019083000

C 3.378283000 1.318627000 -0.085392000

H 2.652224000 2.105290000 -0.253535000

C 4.726806000 1.622464000 0.043725000

H 5.063915000 2.651905000 -0.004668000

C 5.635199000 0.583346000 0.222205000

H 6.697372000 0.770863000 0.323145000

C 5.138668000 -0.717945000 0.261882000

H 5.814153000 -1.559162000 0.394077000

N -0.617783000 -0.422266000 -0.154493000

N -0.169620000 -1.665620000 -0.488000000

N 1.121366000 -1.640452000 -0.470403000

N 3.846925000 -1.025390000 0.150709000

# L^2^ S = 0 *trans* isomer

C -2.005297000 -0.161275000 -0.039750000

C -2.479751000 1.100002000 -0.393014000

H -1.795570000 1.870129000 -0.727049000

C -3.846236000 1.359807000 -0.340984000

H -4.205036000 2.344740000 -0.619803000

C -4.760143000 0.376382000 0.044360000

C -4.258653000 -0.887833000 0.378023000

H -4.945510000 -1.673578000 0.674946000

C -2.898130000 -1.162170000 0.346422000

H -2.515662000 -2.139490000 0.606581000

C -6.239574000 0.664798000 0.114940000

H -6.569258000 0.776465000 1.153366000

H -6.824431000 -0.146995000 -0.325362000

H -6.491453000 1.587037000 -0.412168000

C 0.439875000 0.408725000 0.049515000

H 0.345244000 1.464695000 0.222207000

C 1.552089000 -0.396846000 -0.059989000

C 2.965140000 -0.017174000 0.003826000

C 3.975850000 -0.979444000 -0.114357000

H 3.706284000 -2.017968000 -0.255121000

C 5.298631000 -0.563740000 -0.046977000

H 6.103869000 -1.284486000 -0.135561000

C 5.573827000 0.789425000 0.135820000

H 6.591191000 1.156994000 0.194105000

C 4.499293000 1.669504000 0.243107000

H 4.671831000 2.732856000 0.386575000

N -0.610314000 -0.441737000 -0.073966000

N -0.165245000 -1.717034000 -0.250836000

N 1.128164000 -1.683975000 -0.239327000

N 3.222677000 1.289694000 0.180077000

# [Mn(L^2^)_2_Cl_2_] S = 5/2 *cct* isomer (R = CH_3_)

Mn 0.005833000 -2.146453000 0.266880000

Cl -1.967921000 -3.523365000 0.492328000

N -0.048220000 -2.122247000 -2.066070000

N -1.485011000 -0.426187000 -0.485296000

N -2.291001000 0.398912000 0.099066000

N -3.126680000 0.876232000 -0.849689000

C 0.706421000 -2.958499000 -2.786616000

H 1.435455000 -3.528561000 -2.219660000

C 0.565198000 -3.092551000 -4.164631000

H 1.195342000 -3.785807000 -4.707090000

C -0.397889000 -2.327488000 -4.813621000

H -0.537966000 -2.407551000 -5.885600000

C -1.184936000 -1.457378000 -4.069084000

H -1.942882000 -0.849944000 -4.548386000

C -0.983642000 -1.382481000 -2.689880000

C -1.767001000 -0.503026000 -1.819667000

C -2.829805000 0.338048000 -2.062299000

H -3.363822000 0.601503000 -2.957304000

Cl 2.038578000 -3.455211000 0.366574000

N 0.016286000 -1.520653000 2.515741000

N 1.455671000 -0.263253000 0.569747000

N 2.276898000 0.377972000 -0.195972000

N 3.102583000 1.081884000 0.609427000

C -0.746715000 -2.148468000 3.416674000

H -1.452150000 -2.863880000 3.006162000

C -0.641168000 -1.905532000 4.783007000

H -1.276424000 -2.440751000 5.477183000

C 0.292624000 -0.974231000 5.224429000

H 0.404244000 -0.761390000 6.281519000

C 1.085978000 -0.317341000 4.291658000

H 1.819442000 0.414729000 4.606870000

C 0.921930000 -0.619254000 2.938806000

C 1.714612000 0.012765000 1.882339000

C 2.783805000 0.880103000 1.915309000

H 3.348668000 1.314998000 2.720079000

C -4.147733000 1.812923000 -0.513021000

C -5.356065000 1.809111000 -1.204212000

C -3.925435000 2.730011000 0.514003000

C -6.336228000 2.740672000 -0.874145000

C -4.920718000 3.641743000 0.838214000

C -6.140584000 3.669714000 0.150504000

H -5.545593000 1.071449000 -1.974347000

H -2.984097000 2.713559000 1.046271000

H -7.275570000 2.730690000 -1.416053000

H -4.742611000 4.351921000 1.638805000

C 4.149378000 1.876925000 0.056906000

C 4.518907000 3.071275000 0.669280000

C 4.794682000 1.446366000 -1.102089000

C 5.552039000 3.828237000 0.124066000

C 5.812305000 2.223148000 -1.639204000

C 6.215782000 3.421243000 -1.035966000

H 3.992415000 3.422247000 1.548732000

H 4.492761000 0.515484000 -1.563011000

H 5.834940000 4.758544000 0.604398000

H 6.311169000 1.885020000 -2.541210000

C -7.215801000 4.658574000 0.527772000

H -8.002841000 4.699484000 -0.227227000

H -7.682433000 4.384741000 1.479632000

H -6.806035000 5.665330000 0.645038000

C 7.345375000 4.234829000 -1.617707000

H 8.311691000 3.757631000 -1.423958000

H 7.244973000 4.335436000 -2.701476000

H 7.378737000 5.236978000 -1.186335000

# [Mn(L^2^)_2_Cl_2_] S = 5/2 *ttt* isomer (R = CH_3_)

Mn 0.000239000 0.000680000 0.000201000

Cl 0.018925000 0.148310000 2.440999000

N 0.216052000 -2.323545000 0.121817000

N 2.265678000 -0.502186000 0.018393000

N 3.357119000 0.189795000 -0.008881000

N 4.381587000 -0.689369000 0.013354000

C -0.820424000 -3.161516000 0.181473000

H -1.802866000 -2.701721000 0.188639000

C -0.669319000 -4.542817000 0.233138000

H -1.540214000 -5.183954000 0.282254000

C 0.618920000 -5.068558000 0.220146000

H 0.778796000 -6.139909000 0.257895000

C 1.701975000 -4.201678000 0.158869000

H 2.715082000 -4.584353000 0.147700000

C 1.462902000 -2.826308000 0.112313000

C 2.544079000 -1.838555000 0.057278000

C 3.915715000 -1.965301000 0.055382000

H 4.572452000 -2.814735000 0.110783000

Cl -0.018178000 -0.146562000 -2.440637000

N -0.216619000 2.324826000 -0.121389000

N -2.265460000 0.502571000 -0.018068000

N -3.356607000 -0.189886000 0.008999000

N -4.381456000 0.688829000 -0.013421000

C 0.819488000 3.163253000 -0.180917000

H 1.802135000 2.703895000 -0.188054000

C 0.667790000 4.544492000 -0.232493000

H 1.538409000 5.186011000 -0.281505000

C -0.620679000 5.069670000 -0.219539000

H -0.781018000 6.140953000 -0.257214000

C -1.703359000 4.202312000 -0.158390000

H -2.716632000 4.584548000 -0.147239000

C -1.463687000 2.827043000 -0.111927000

C -2.544438000 1.838815000 -0.057031000

C -3.916130000 1.964962000 -0.055367000

H -4.573232000 2.814106000 -0.110901000

C 5.732641000 -0.231764000 0.005819000

C 6.700859000 -0.927380000 -0.711957000

C 6.070308000 0.915071000 0.723790000

C 8.017754000 -0.476138000 -0.697129000

C 7.385978000 1.357735000 0.714260000

C 8.383880000 0.671667000 0.009924000

H 6.428363000 -1.797107000 -1.297610000

H 5.304912000 1.440335000 1.279447000

H 8.768131000 -1.021321000 -1.259048000

H 7.644848000 2.249378000 1.275459000

C -5.732315000 0.230638000 -0.006171000

C -6.700936000 0.925700000 0.711600000

C -6.069386000 -0.916188000 -0.724432000

C -8.017636000 0.473901000 0.696480000

C -7.384871000 -1.359414000 -0.715193000

C -8.383167000 -0.673918000 -0.010862000

H -6.428895000 1.795431000 1.297458000

H -5.303691000 -1.440998000 -1.280108000

H -8.768332000 1.018657000 1.258386000

H -7.643283000 -2.251039000 -1.276632000

C -9.805643000 -1.176838000 -0.008053000

H -10.160780000 -1.365872000 -1.024848000

H -9.887418000 -2.118258000 0.544551000

H -10.482201000 -0.458142000 0.457567000

C 9.806580000 1.173955000 0.006860000

H 10.162247000 1.362063000 1.023646000

H 10.482613000 0.455298000 -0.459581000

H 9.888539000 2.115752000 -0.545065000

# [Fe(L^2^)_2_Cl_2_] S = 2 *cct* isomer (R = CH_3_)

Fe -0.019409000 -2.166816000 0.162471000

Cl -1.994441000 -3.520992000 0.305483000

N -0.152099000 -2.224893000 -2.080672000

N -1.432241000 -0.477959000 -0.449130000

N -2.137766000 0.409870000 0.172054000

N -2.937191000 0.991557000 -0.747923000

C 0.514237000 -3.114500000 -2.823803000

H 1.225727000 -3.727533000 -2.281096000

C 0.313174000 -3.241819000 -4.194485000

H 0.873699000 -3.978834000 -4.755457000

C -0.619773000 -2.416274000 -4.813547000

H -0.805060000 -2.491553000 -5.878941000

C -1.318372000 -1.493135000 -4.045366000

H -2.054837000 -0.840390000 -4.497988000

C -1.058674000 -1.426235000 -2.676011000

C -1.743741000 -0.494450000 -1.778957000

C -2.720209000 0.455114000 -1.979136000

H -3.239200000 0.795101000 -2.856906000

Cl 1.976176000 -3.482459000 0.227958000

N 0.063813000 -1.836080000 2.385563000

N 1.389149000 -0.391073000 0.503657000

N 2.137476000 0.354368000 -0.242237000

N 2.932569000 1.066734000 0.584929000

C -0.626083000 -2.579306000 3.257017000

H -1.319983000 -3.284379000 2.811764000

C -0.469589000 -2.452164000 4.633635000

H -1.046602000 -3.078660000 5.301915000

C 0.439041000 -1.518149000 5.120485000

H 0.588139000 -1.394797000 6.187098000

C 1.158708000 -0.743584000 4.219161000

H 1.874300000 -0.009067000 4.568114000

C 0.946720000 -0.932654000 2.852927000

C 1.664093000 -0.177135000 1.824619000

C 2.668776000 0.762474000 1.884066000

H 3.223307000 1.189582000 2.700063000

C -3.852864000 2.015776000 -0.366105000

C -5.057427000 2.168628000 -1.047149000

C -3.532677000 2.859215000 0.697507000

C -5.934100000 3.181365000 -0.668598000

C -4.426657000 3.853966000 1.069505000

C -5.639829000 4.038408000 0.394528000

H -5.325812000 1.490369000 -1.847852000

H -2.596384000 2.721873000 1.220959000

H -6.870895000 3.293278000 -1.203366000

H -4.172341000 4.505924000 1.898565000

C 3.906326000 1.965089000 0.057903000

C 4.222234000 3.135319000 0.741893000

C 4.540124000 1.656348000 -1.145914000

C 5.190751000 3.990811000 0.223393000

C 5.491855000 2.529488000 -1.654073000

C 5.841126000 3.706846000 -0.979497000

H 3.705553000 3.391655000 1.659031000

H 4.282314000 0.741474000 -1.662290000

H 5.432629000 4.900902000 0.761339000

H 5.982705000 2.283888000 -2.589902000

C -6.607367000 5.112624000 0.825703000

H -7.362023000 5.301606000 0.059994000

H -7.131017000 4.821531000 1.742303000

H -6.090537000 6.053460000 1.031796000

C 6.891673000 4.632598000 -1.541349000

H 7.002638000 5.528533000 -0.928159000

H 7.866388000 4.137480000 -1.590309000

H 6.637607000 4.948792000 -2.557207000

# [Fe(L^2^)_2_Cl_2_] S = 2 *ttt* isomer (R = CH_3_)

Fe -0.000002000 -0.000060000 0.001262000

Cl -0.031543000 -0.147369000 2.396962000

N -0.115963000 2.247153000 0.123042000

N -2.186300000 0.490317000 0.010692000

N -3.285918000 -0.188358000 -0.018308000

N -4.299447000 0.703401000 0.006556000

C 0.948093000 3.048684000 0.187166000

H 1.912778000 2.554011000 0.193680000

C 0.840257000 4.434221000 0.243651000

H 1.731082000 5.047182000 0.296506000

C -0.429827000 5.002447000 0.230523000

H -0.554048000 6.078430000 0.271821000

C -1.541474000 4.172275000 0.164490000

H -2.541869000 4.587226000 0.153081000

C -1.345807000 2.790982000 0.113489000

C -2.448954000 1.829710000 0.053621000

C -3.818101000 1.974248000 0.051950000

H -4.464772000 2.831282000 0.109945000

Cl 0.031558000 0.147302000 -2.394678000

N 0.115959000 -2.247233000 -0.121049000

N 2.186312000 -0.490388000 -0.008942000

N 3.285930000 0.188315000 0.019368000

N 4.299470000 -0.703390000 -0.006939000

C -0.948112000 -3.048778000 -0.184763000

H -1.912810000 -2.554124000 -0.190723000

C -0.840273000 -4.434302000 -0.241554000

H -1.731111000 -5.047273000 -0.294066000

C 0.429831000 -5.002501000 -0.229161000

H 0.554054000 -6.078475000 -0.270690000

C 1.541491000 -4.172317000 -0.163543000

H 2.541901000 -4.587249000 -0.152670000

C 1.345820000 -2.791036000 -0.112226000

C 2.448978000 -1.829750000 -0.052796000

C 3.818132000 -1.974233000 -0.052510000

H 4.464783000 -2.831207000 -0.111611000

C -5.656357000 0.264083000 0.000377000

C -6.614048000 0.968869000 -0.723452000

C -6.010504000 -0.875448000 0.721265000

C -7.936462000 0.535478000 -0.709373000

C -7.332365000 -1.300838000 0.710903000

C -8.320005000 -0.602687000 0.004905000

H -6.327448000 1.829347000 -1.316004000

H -5.252645000 -1.411001000 1.277488000

H -8.677618000 1.085180000 -1.279176000

H -7.603220000 -2.189498000 1.271047000

C 5.656367000 -0.264015000 -0.001958000

C 6.614827000 -0.969060000 0.720618000

C 6.009732000 0.875814000 -0.722741000

C 7.937203000 -0.535623000 0.705357000

C 7.331595000 1.301253000 -0.713554000

C 8.319975000 0.602854000 -0.008860000

H 6.328853000 -1.829786000 1.313113000

H 5.251288000 1.411561000 -1.277981000

H 8.678970000 -1.085523000 1.274176000

H 7.601841000 2.190151000 -1.273611000

C -9.757363000 -1.060589000 0.031799000

H -10.334815000 -0.609384000 -0.777249000

H -9.830444000 -2.146730000 -0.064261000

H -10.238303000 -0.784181000 0.976086000

C 9.757301000 1.060784000 -0.037004000

H 10.335509000 0.609438000 0.771428000

H 10.237365000 0.784539000 -0.981787000

H 9.830472000 2.146906000 0.059171000

# [Co(L^2^)_2_Cl_2_] S = 3/2 *cct* isomer (R = CH_3_)

Co -0.011404000 -2.026469000 0.145709000

Cl -1.937059000 -3.462722000 0.213624000

N -0.082995000 -2.142169000 -2.053320000

N -1.404699000 -0.437596000 -0.475726000

N -2.132562000 0.427652000 0.151123000

N -2.959237000 0.977801000 -0.763151000

C 0.614611000 -3.033640000 -2.763407000

H 1.334276000 -3.612563000 -2.194421000

C 0.426957000 -3.205634000 -4.131893000

H 1.012671000 -3.942646000 -4.666481000

C -0.522831000 -2.425869000 -4.782516000

H -0.696428000 -2.537597000 -5.846689000

C -1.255661000 -1.502073000 -4.046894000

H -2.008546000 -0.884968000 -4.521930000

C -1.009679000 -1.391446000 -2.679068000

C -1.726352000 -0.469037000 -1.801265000

C -2.734515000 0.446713000 -1.996118000

H -3.272701000 0.766187000 -2.870016000

Cl 1.958050000 -3.398648000 0.277602000

N 0.031373000 -1.796828000 2.334522000

N 1.368401000 -0.348212000 0.525956000

N 2.120569000 0.394931000 -0.218016000

N 2.939466000 1.075442000 0.611718000

C -0.675098000 -2.567484000 3.166985000

H -1.376863000 -3.240034000 2.685171000

C -0.517750000 -2.508641000 4.548731000

H -1.108370000 -3.155222000 5.185189000

C 0.407408000 -1.617786000 5.081649000

H 0.556376000 -1.549453000 6.153222000

C 1.146092000 -0.816036000 4.219714000

H 1.878029000 -0.115344000 4.602653000

C 0.933149000 -0.937722000 2.847219000

C 1.662546000 -0.163630000 1.845592000

C 2.684832000 0.755211000 1.909567000

H 3.256974000 1.157489000 2.726005000

C -3.906137000 1.971426000 -0.376196000

C -5.123778000 2.075254000 -1.043014000

C -3.603130000 2.833822000 0.677109000

C -6.031688000 3.058494000 -0.660461000

C -4.527789000 3.798472000 1.053454000

C -5.755191000 3.933654000 0.392696000

H -5.377259000 1.381288000 -1.835043000

H -2.655966000 2.734363000 1.189503000

H -6.978632000 3.132608000 -1.183844000

H -4.287007000 4.465568000 1.874445000

C 3.925846000 1.962206000 0.088227000

C 4.268844000 3.117225000 0.784911000

C 4.543716000 1.657554000 -1.124746000

C 5.248706000 3.961461000 0.269440000

C 5.507215000 2.519728000 -1.629575000

C 5.883807000 3.681301000 -0.942554000

H 3.764203000 3.370874000 1.709466000

H 4.264727000 0.754370000 -1.650624000

H 5.511694000 4.859943000 0.816909000

H 5.985637000 2.277783000 -2.572740000

C -6.755385000 4.975557000 0.828538000

H -7.527604000 5.129216000 0.072533000

H -7.254354000 4.674206000 1.755515000

H -6.270813000 5.936788000 1.018319000

C 6.949101000 4.593146000 -1.499349000

H 7.055412000 5.496725000 -0.896623000

H 7.921567000 4.091520000 -1.524206000

H 6.714749000 4.896350000 -2.523694000

# [Co(L^2^)_2_Cl_2_] S = 3/2 *ttt* isomer (R = CH_3_)

Co -0.000010000 -0.000013000 0.000917000

Cl -0.024429000 -0.155712000 2.415065000

N -0.016477000 2.182051000 0.126337000

N -2.101403000 0.504370000 0.017312000

N -3.206750000 -0.164644000 -0.011068000

N -4.210416000 0.736977000 0.010965000

C 1.077037000 2.944069000 0.192992000

H 2.023300000 2.415978000 0.199512000

C 1.018673000 4.332211000 0.251813000

H 1.932070000 4.910687000 0.306564000

C -0.228750000 4.948478000 0.238918000

H -0.312400000 6.028230000 0.282085000

C -1.370089000 4.160217000 0.171139000

H -2.355302000 4.609932000 0.160461000

C -1.224797000 2.773792000 0.117523000

C -2.350320000 1.844999000 0.056867000

C -3.717455000 2.003415000 0.054051000

H -4.355493000 2.867004000 0.109657000

Cl 0.024413000 0.155648000 -2.413332000

N 0.016462000 -2.182094000 -0.124557000

N 2.101387000 -0.504361000 -0.015912000

N 3.206731000 0.164687000 0.011833000

N 4.210411000 -0.736893000 -0.011115000

C -1.077047000 -2.944151000 -0.190834000

H -2.023326000 -2.416087000 -0.197106000

C -1.018660000 -4.332293000 -0.249612000

H -1.932056000 -4.910800000 -0.304057000

C 0.228785000 -4.948523000 -0.237058000

H 0.312454000 -6.028276000 -0.280181000

C 1.370120000 -4.160224000 -0.169670000

H 2.355350000 -4.609908000 -0.159241000

C 1.224803000 -2.773799000 -0.116096000

C 2.350322000 -1.844973000 -0.055904000

C 3.717465000 -2.003340000 -0.054120000

H 4.355491000 -2.866892000 -0.110427000

C -5.571597000 0.310184000 0.005852000

C -6.518233000 1.010589000 -0.735795000

C -5.941212000 -0.808270000 0.751863000

C -7.845737000 0.592276000 -0.717570000

C -7.267522000 -1.218801000 0.745649000

C -8.243843000 -0.527703000 0.016723000

H -6.220367000 1.858298000 -1.341088000

H -5.193058000 -1.335378000 1.329058000

H -8.579425000 1.141057000 -1.297757000

H -7.551914000 -2.087773000 1.329542000

C 5.571585000 -0.310060000 -0.007162000

C 6.518913000 -1.010537000 0.733564000

C 5.940529000 0.808366000 -0.753516000

C 7.846407000 -0.592319000 0.714034000

C 7.266892000 1.218813000 -0.748608000

C 8.243864000 0.527666000 -0.020643000

H 6.221601000 -1.858297000 1.339058000

H 5.191861000 1.335460000 -1.330056000

H 8.580652000 -1.141208000 1.293421000

H 7.550792000 2.087701000 -1.332854000

C -9.677567000 -0.997532000 0.012736000

H -10.023480000 -1.223921000 1.024734000

H -10.343465000 -0.244214000 -0.411877000

H -9.788130000 -1.911146000 -0.580484000

C 9.677559000 0.997583000 -0.017571000

H 10.019908000 1.233012000 -1.028678000

H 9.790106000 1.905888000 0.583430000

H 10.345006000 0.240645000 0.398112000

# [Ni(L^2^)_2_Cl_2_] S = 1 *cct* isomer (R = CH_3_)

Ni 0.003607000 -1.883642000 0.173127000

Cl -1.822492000 -3.448940000 0.071750000

N 0.150520000 -1.965698000 -1.945391000

N -1.433688000 -0.411556000 -0.450493000

N -2.292208000 0.360375000 0.129413000

N -3.067272000 0.882695000 -0.844663000

C 0.972846000 -2.785792000 -2.604792000

H 1.652939000 -3.353105000 -1.977662000

C 0.941919000 -2.905529000 -3.991352000

H 1.621956000 -3.586493000 -4.487083000

C 0.023886000 -2.148770000 -4.710872000

H -0.027660000 -2.221838000 -5.791178000

C -0.837865000 -1.300222000 -4.025494000

H -1.570434000 -0.704797000 -4.556474000

C -0.748559000 -1.238306000 -2.636211000

C -1.616796000 -0.408308000 -1.803468000

C -2.674739000 0.429999000 -2.066763000

H -3.149723000 0.742340000 -2.978979000

Cl 1.882127000 -3.345210000 0.553656000

N -0.157792000 -1.567324000 2.267890000

N 1.412324000 -0.295557000 0.515560000

N 2.270029000 0.358853000 -0.195429000

N 3.043053000 1.051603000 0.667495000

C -0.984193000 -2.250906000 3.063636000

H -1.655967000 -2.930634000 2.549492000

C -0.966707000 -2.103856000 4.447853000

H -1.650011000 -2.680528000 5.058084000

C -0.057879000 -1.219598000 5.017951000

H -0.015698000 -1.086471000 6.092945000

C 0.805068000 -0.509255000 4.191112000

H 1.527828000 0.183022000 4.605305000

C 0.728249000 -0.712407000 2.814652000

C 1.591120000 -0.040624000 1.844969000

C 2.651256000 0.829379000 1.951602000

H 3.167172000 1.254531000 2.793502000

C -4.130750000 1.776909000 -0.523588000

C -5.274875000 1.814483000 -1.317162000

C -4.015936000 2.610764000 0.587353000

C -6.296790000 2.703990000 -1.002452000

C -5.053803000 3.481714000 0.893889000

C -6.209131000 3.550009000 0.106863000

H -5.382904000 1.139888000 -2.157623000

H -3.124077000 2.564613000 1.197311000

H -7.184540000 2.726748000 -1.625135000

H -4.958758000 4.127758000 1.760070000

C 4.115474000 1.860755000 0.189326000

C 4.436162000 3.050211000 0.837638000

C 4.835683000 1.448931000 -0.931580000

C 5.495439000 3.821087000 0.367451000

C 5.878946000 2.239456000 -1.394367000

C 6.234457000 3.432857000 -0.752894000

H 3.852909000 3.386864000 1.686360000

H 4.571396000 0.521344000 -1.421433000

H 5.739456000 4.747753000 0.875406000

C -7.339634000 4.482152000 0.466162000

H -7.917740000 4.767043000 -0.415298000

H -8.028869000 4.004929000 1.171113000

H -6.968999000 5.394326000 0.939007000

H 6.435720000 1.915620000 -2.267265000

C 7.392972000 4.260533000 -1.251712000

H 8.348260000 3.793247000 -0.990747000

H 7.368558000 4.363061000 -2.339605000

H 7.384737000 5.261619000 -0.816759000

# [Ni(L^2^)_2_Cl_2_] S = 1 *ttt* isomer (R = CH_3_)

Ni 0.000014000 -0.000013000 0.000844000

Cl 0.030646000 0.162110000 2.423495000

N -0.054769000 -2.127560000 0.129522000

N 2.050520000 -0.484586000 0.012874000

N 3.162737000 0.171741000 -0.015497000

N 4.155140000 -0.740877000 0.007911000

C -1.159125000 -2.872875000 0.202904000

H -2.097039000 -2.331827000 0.212407000

C -1.120309000 -4.261838000 0.264466000

H -2.042672000 -4.825308000 0.324544000

C 0.116517000 -4.897799000 0.247589000

H 0.183932000 -5.978571000 0.292545000

C 1.269053000 -4.126513000 0.173892000

H 2.247734000 -4.590046000 0.160538000

C 1.144381000 -2.738969000 0.117957000

C 2.283116000 -1.827571000 0.053844000

C 3.647896000 -2.001637000 0.051887000

H 4.275895000 -2.872388000 0.109116000

Cl -0.030624000 -0.162154000 -2.421890000

N 0.054798000 2.127542000 -0.127821000

N -2.050503000 0.484533000 -0.011575000

N -3.162724000 -0.171821000 0.016122000

N -4.155131000 0.740770000 -0.008075000

C 1.159163000 2.872890000 -0.200759000

H 2.097090000 2.331861000 -0.210058000

C 1.120339000 4.261858000 -0.262171000

H 2.042710000 4.825354000 -0.321893000

C -0.116503000 4.897795000 -0.245595000

H -0.183924000 5.978572000 -0.290427000

C -1.269047000 4.126477000 -0.172351000

H -2.247740000 4.589990000 -0.159211000

C -1.144365000 2.738928000 -0.116559000

C -2.283106000 1.827504000 -0.052947000

C -3.647891000 2.001533000 -0.051998000

H -4.275871000 2.872254000 -0.109896000

C 5.521292000 -0.329551000 0.004829000

C 6.458248000 -1.034152000 -0.745073000

C 5.904977000 0.776514000 0.761849000

C 7.790669000 -0.632190000 -0.724308000

C 7.236252000 1.171025000 0.757991000

C 8.203068000 0.475687000 0.020610000

H 6.149078000 -1.872239000 -1.358060000

H 5.164288000 1.305879000 1.346665000

H 8.517030000 -1.184136000 -1.310674000

H 7.532072000 2.030098000 1.350722000

C -5.521282000 0.329432000 -0.006095000

C -6.458895000 1.034162000 0.742921000

C -5.904351000 -0.776593000 -0.763427000

C -7.791312000 0.632370000 0.720882000

C -7.235708000 -1.170957000 -0.760838000

C -8.203128000 -0.475509000 -0.024423000

H -6.150221000 1.872293000 1.356101000

H -5.163188000 -1.305999000 -1.347604000

H -8.518197000 1.184468000 1.306464000

H -7.531078000 -2.029948000 -1.353901000

C -9.642080000 -0.929029000 -0.021834000

H -9.978034000 -1.188883000 -1.028901000

H -9.770051000 -1.818766000 0.603538000

H -10.304534000 -0.153598000 0.366951000

C 9.641993000 0.929293000 0.017092000

H 9.981523000 1.179721000 1.025389000

H 10.303072000 0.157535000 -0.381181000

H 9.767735000 1.824872000 -0.600296000

# [Cu(L^2^)_2_Cl_2_] S = 1/2 *cct* isomer (R = CH_3_)

Cu -0.216218000 -1.862896000 0.382204000

Cl 1.626161000 -3.185381000 0.875638000

N -0.116359000 -1.053517000 2.248269000

N 1.500797000 0.087701000 0.224088000

N 2.472525000 0.551889000 -0.488638000

N 3.401452000 1.050935000 0.366550000

C -0.985053000 -1.491919000 3.170131000

H -1.718604000 -2.208182000 2.806399000

C -0.939165000 -1.061302000 4.490458000

H -1.654950000 -1.448771000 5.203887000

C 0.034638000 -0.142054000 4.861669000

H 0.098335000 0.217438000 5.882460000

C 0.927356000 0.317619000 3.902636000

H 1.683780000 1.048993000 4.157561000

C 0.835513000 -0.162376000 2.594744000

C 1.763358000 0.276713000 1.551301000

C 2.989263000 0.898419000 1.650308000

H 3.574850000 1.228809000 2.489178000

Cl -2.453605000 -3.222047000 0.744818000

N -0.322732000 -2.443333000 -1.594745000

N -1.551291000 -0.428598000 -0.380094000

N -2.261382000 0.534803000 0.103209000

N -3.108049000 0.916346000 -0.875306000

C 0.328725000 -3.490089000 -2.109490000

H 1.023189000 -3.978286000 -1.436238000

C 0.122876000 -3.918016000 -3.416705000

H 0.669204000 -4.773572000 -3.791948000

C -0.790182000 -3.235280000 -4.211842000

H -0.974437000 -3.545523000 -5.233833000

C -1.473181000 -2.149938000 -3.676436000

H -2.197201000 -1.601785000 -4.266135000

C -1.220615000 -1.783400000 -2.357330000

C -1.897959000 -0.689819000 -1.671928000

C -2.912834000 0.177368000 -2.001218000

H -3.518951000 0.296049000 -2.881115000

C 4.609983000 1.622657000 -0.125868000

C 5.787045000 1.518808000 0.610211000

C 4.603886000 2.284078000 -1.354258000

C 6.955164000 2.096039000 0.119685000

C 5.781804000 2.840400000 -1.833518000

C 6.977031000 2.763367000 -1.107043000

H 5.803678000 0.969798000 1.543768000

H 3.682865000 2.347674000 -1.917611000

H 7.868165000 2.008937000 0.698496000

H 5.770123000 3.352144000 -2.790221000

C -4.065905000 1.947524000 -0.642456000

C -4.389095000 2.841882000 -1.658677000

C -4.672834000 2.045690000 0.609010000

C -5.339886000 3.830376000 -1.421216000

C -5.607493000 3.048029000 0.830790000

C -5.964850000 3.952330000 -0.177534000

H -3.889229000 2.785206000 -2.618292000

H -4.409800000 1.338877000 1.384565000

H -5.587398000 4.526060000 -2.215459000

H -6.077845000 3.121162000 1.805563000

C 8.248628000 3.367483000 -1.649861000

H 9.047988000 3.348501000 -0.907054000

H 8.598782000 2.819256000 -2.530295000

H 8.095293000 4.406487000 -1.954663000

C -7.006891000 5.014152000 0.073207000

H -7.001674000 5.771307000 -0.712932000

H -8.010198000 4.576916000 0.106946000

H -6.839808000 5.516753000 1.029360000

# [Cu(L^2^)_2_Cl_2_] S = 1/2 *ttt* isomer (R = CH_3_)

Cu 0.000004000 -0.000008000 -0.000137000

Cl 0.049129000 -0.151585000 -2.351758000

N -0.111851000 2.455347000 -0.136334000

N 1.925993000 0.648220000 -0.008788000

N 3.011040000 -0.054173000 0.018968000

N 4.034125000 0.818773000 0.000347000

C -1.151569000 3.283193000 -0.206750000

H -2.127887000 2.810639000 -0.232888000

C -1.013298000 4.668646000 -0.247293000

H -1.888472000 5.303861000 -0.306410000

C 0.269423000 5.207075000 -0.210515000

H 0.419572000 6.280250000 -0.238573000

C 1.359886000 4.349260000 -0.138225000

H 2.369828000 4.740324000 -0.108351000

C 1.123887000 2.972477000 -0.105246000

C 2.204818000 1.983701000 -0.043824000

C 3.576253000 2.098949000 -0.039224000

H 4.238177000 2.944319000 -0.093101000

Cl -0.049114000 0.151555000 2.351469000

N 0.111832000 -2.455405000 0.135841000

N -1.925985000 -0.648228000 0.008533000

N -3.011024000 0.054190000 -0.018920000

N -4.034122000 -0.818737000 -0.000225000

C 1.151540000 -3.283285000 0.205989000

H 2.127862000 -2.810748000 0.232307000

C 1.013257000 -4.668750000 0.246053000

H 1.888424000 -5.303994000 0.304966000

C -0.269468000 -5.207155000 0.209060000

H -0.419626000 -6.280339000 0.236736000

C -1.359921000 -4.349306000 0.137041000

H -2.369866000 -4.740351000 0.106985000

C -1.123909000 -2.972513000 0.104548000

C -2.204831000 -1.983709000 0.043433000

C -3.576269000 -2.098929000 0.039074000

H -4.238198000 -2.944291000 0.092984000

C 5.383511000 0.354864000 -0.000781000

C 6.350571000 1.026268000 0.740974000

C 5.720158000 -0.768637000 -0.754599000

C 7.666462000 0.573006000 0.715486000

C 7.035207000 -1.214048000 -0.755565000

C 8.032029000 -0.552985000 -0.026476000

H 6.077302000 1.878394000 1.351627000

H 4.955890000 -1.271700000 -1.332234000

H 8.416615000 1.099022000 1.295712000

H 7.294476000 -2.086724000 -1.345596000

C -5.383499000 -0.354800000 0.001303000

C -6.350749000 -1.026050000 -0.740353000

C -5.719953000 0.768537000 0.755440000

C -7.666628000 -0.572797000 -0.714438000

C -7.035009000 1.213948000 0.756835000

C -8.032009000 0.553045000 0.027857000

H -6.077635000 -1.878063000 -1.351235000

H -4.955545000 1.271465000 1.333008000

H -8.416933000 -1.098701000 -1.294569000

H -7.294133000 2.086481000 1.347137000

C -9.452933000 1.060277000 0.032382000

H -9.787435000 1.293631000 1.046610000

H -9.542644000 1.977657000 -0.558568000

H -10.140545000 0.325485000 -0.390132000

C 9.452971000 -1.060173000 -0.030666000

H 9.788392000 -1.291589000 -1.045045000

H 10.140180000 -0.326153000 0.393831000

H 9.542182000 -1.978675000 0.558603000

# [Zn(L^2^)_2_Cl_2_] S = 0 *cct* isomer (R = CH_3_)

Zn 0.009992000 -2.100208000 0.182112000

Cl -1.902267000 -3.516645000 0.149409000

N 0.131023000 -1.968562000 -2.016966000

N -1.447778000 -0.407408000 -0.456296000

N -2.308200000 0.363179000 0.121716000

N -3.063792000 0.915298000 -0.854284000

C 0.953505000 -2.754848000 -2.717248000

H 1.625512000 -3.364612000 -2.121811000

C 0.941638000 -2.789514000 -4.108266000

H 1.621183000 -3.445479000 -4.637142000

C 0.041719000 -1.976554000 -4.788786000

H 0.003335000 -1.980017000 -5.872141000

C -0.815126000 -1.158815000 -4.062017000

H -1.527340000 -0.516750000 -4.565532000

C -0.744802000 -1.182911000 -2.668948000

C -1.610071000 -0.373221000 -1.810535000

C -2.655771000 0.481542000 -2.076653000

H -3.112158000 0.816183000 -2.990521000

Cl 1.980340000 -3.416163000 0.447768000

N -0.135021000 -1.583716000 2.321817000

N 1.424443000 -0.292409000 0.517294000

N 2.282780000 0.369980000 -0.184801000

N 3.033959000 1.085723000 0.681878000

C -0.958784000 -2.239769000 3.143934000

H -1.617117000 -2.954812000 2.660898000

C -0.963766000 -2.021684000 4.518362000

H -1.643818000 -2.578332000 5.150316000

C -0.079986000 -1.088529000 5.049328000

H -0.054133000 -0.896343000 6.115920000

C 0.775435000 -0.402878000 4.195163000

H 1.472625000 0.330697000 4.581053000

C 0.721800000 -0.678854000 2.828804000

C 1.581118000 -0.019851000 1.844863000

C 2.626476000 0.868788000 1.960615000

H 3.122944000 1.309645000 2.806219000

C -4.123337000 1.813173000 -0.531902000

C -5.251852000 1.883982000 -1.345194000

C -4.020698000 2.617662000 0.601948000

C -6.269922000 2.776978000 -1.027083000

C -5.054402000 3.492383000 0.911056000

C -6.193987000 3.593668000 0.104556000

H -5.351535000 1.232247000 -2.204531000

H -3.141104000 2.545147000 1.226903000

H -7.145456000 2.825705000 -1.665375000

H -4.968775000 4.115406000 1.794967000

C 4.101081000 1.905307000 0.210553000

C 4.399233000 3.104012000 0.852577000

C 4.839898000 1.493900000 -0.898494000

C 5.454474000 3.884383000 0.388785000

C 5.878695000 2.293664000 -1.355252000

C 6.211752000 3.496511000 -0.719338000

H 3.801805000 3.440219000 1.691582000

H 4.592903000 0.559244000 -1.383889000

H 5.680772000 4.818054000 0.892125000

H 6.449840000 1.969750000 -2.218821000

C -7.319979000 4.530165000 0.466777000

H -7.901835000 4.813827000 -0.412495000

H -8.006563000 4.057653000 1.177436000

H -6.944092000 5.442995000 0.934515000

C 7.365468000 4.334672000 -1.211754000

H 8.323526000 3.874442000 -0.948543000

H 7.344212000 4.439674000 -2.299553000

H 7.347527000 5.334656000 -0.774573000

# [Zn(L^2^)_2_Cl_2_] S = 0 *ttt* isomer (R = CH_3_)

Zn -0.000001000 0.000100000 0.001022000

Cl -0.030589000 0.156930000 -2.418981000

N -0.087065000 -2.207855000 -0.125416000

N -2.176181000 -0.465034000 -0.009519000

N -3.280030000 0.204922000 0.020217000

N -4.285016000 -0.695537000 -0.006051000

C 0.982871000 -3.001433000 -0.192279000

H 1.944844000 -2.501729000 -0.199161000

C 0.882955000 -4.387006000 -0.250970000

H 1.777711000 -4.993795000 -0.305786000

C -0.383682000 -4.962697000 -0.237609000

H -0.501623000 -6.039252000 -0.280542000

C -1.499960000 -4.139326000 -0.169431000

H -2.497753000 -4.560155000 -0.158144000

C -1.313599000 -2.757211000 -0.116016000

C -2.424420000 -1.805057000 -0.054489000

C -3.792509000 -1.961569000 -0.053198000

H -4.431512000 -2.824165000 -0.112580000

Cl 0.030396000 -0.156553000 2.420816000

N 0.087226000 2.208098000 0.127071000

N 2.176132000 0.465118000 0.010814000

N 3.279915000 -0.204942000 -0.019423000

N 4.284969000 0.695456000 0.005645000

C -0.982634000 3.001741000 0.194323000

H -1.944625000 2.502079000 0.201916000

C -0.882620000 4.387329000 0.252497000

H -1.777314000 4.994179000 0.307647000

C 0.384037000 4.962956000 0.238204000

H 0.502051000 6.039519000 0.280721000

C 1.500238000 4.139511000 0.169645000

H 2.498046000 4.560286000 0.157664000

C 1.313776000 2.757391000 0.116766000

C 2.424502000 1.805142000 0.054916000

C 3.792601000 1.961553000 0.052562000

H 4.431738000 2.824119000 0.110950000

C -5.646210000 -0.268288000 -0.001237000

C -6.594432000 -0.972308000 0.735408000

C -6.012742000 0.857756000 -0.736718000

C -7.920888000 -0.551568000 0.719620000

C -7.338546000 1.270747000 -0.727986000

C -8.317219000 0.572999000 -0.009131000

H -6.297397000 -1.821663000 1.338790000

H -5.261888000 1.392247000 -1.303476000

H -8.655039000 -1.100142000 1.299440000

H -7.619645000 2.148860000 -1.299554000

C 5.646141000 0.268115000 -0.000002000

C 6.593683000 0.971275000 -0.738268000

C 6.013300000 -0.857147000 0.736420000

C 7.920164000 0.550461000 -0.723251000

C 7.339041000 -1.270206000 0.726925000

C 8.317091000 -0.573326000 0.006307000

H 6.296137000 1.820042000 -1.342226000

H 5.262953000 -1.390919000 1.304527000

H 8.653822000 1.098405000 -1.304279000

H 7.620632000 -2.147650000 1.299293000

C 9.758562000 -1.017980000 0.033651000

H 10.331220000 -0.564530000 -0.777481000

H 10.237519000 -0.733967000 0.976649000

H 9.841295000 -2.103786000 -0.058537000

C -9.758870000 1.016966000 -0.037794000

H -10.329652000 0.571498000 0.779096000

H -9.841830000 2.103576000 0.043464000

H -10.239796000 0.723039000 -0.976778000

# [Cd(L^2^)_2_Cl_2_] S = 0 *cct* isomer (R = CH_3_)

Cd -0.001256000 -2.053546000 0.303406000

Cl -2.110212000 -3.271950000 0.864398000

N -0.344060000 -2.064929000 -2.176601000

N -1.555444000 -0.178681000 -0.562057000

N -2.299655000 0.689826000 0.039461000

N -3.258896000 1.067690000 -0.836354000

C 0.323189000 -2.942911000 -2.929398000

H 1.102286000 -3.497117000 -2.415161000

C 0.039280000 -3.137295000 -4.278297000

H 0.601906000 -3.863131000 -4.851576000

C -0.977868000 -2.385355000 -4.857074000

H -1.229627000 -2.509348000 -5.904221000

C -1.670511000 -1.468119000 -4.076491000

H -2.461128000 -0.864570000 -4.505276000

C -1.326245000 -1.334506000 -2.728536000

C -2.002308000 -0.388504000 -1.834696000

C -3.104710000 0.417283000 -2.019410000

H -3.753462000 0.586844000 -2.859654000

Cl 2.139393000 -3.326841000 0.091228000

N 0.270517000 -1.285448000 2.668327000

N 1.534013000 0.008570000 0.577063000

N 2.317655000 0.625240000 -0.244887000

N 3.255521000 1.257284000 0.497167000

C -0.424416000 -1.882737000 3.639728000

H -1.174746000 -2.592025000 3.304457000

C -0.202533000 -1.616839000 4.988103000

H -0.784952000 -2.128167000 5.743961000

C 0.777258000 -0.690385000 5.329418000

H 0.979014000 -0.456686000 6.368641000

C 1.495932000 -0.061823000 4.320042000

H 2.256017000 0.672236000 4.558129000

C 1.217212000 -0.388784000 2.990076000

C 1.930838000 0.223949000 1.865063000

C 3.047380000 1.030858000 1.820563000

H 3.709784000 1.405475000 2.580141000

C -4.249597000 2.021688000 -0.460976000

C -5.534273000 1.942757000 -0.992338000

C -3.921186000 3.032277000 0.441954000

C -6.485143000 2.891100000 -0.627922000

C -4.888126000 3.960437000 0.804950000

C -6.184004000 3.912440000 0.276902000

H -5.801890000 1.135591000 -1.663216000

H -2.920408000 3.075361000 0.849757000

H -7.483767000 2.822489000 -1.045474000

H -4.626769000 4.744012000 1.508252000

C 4.293287000 2.005963000 -0.131303000

C 4.788956000 3.159210000 0.470178000

C 4.805183000 1.569352000 -1.353266000

C 5.814513000 3.867661000 -0.150016000

C 5.816959000 2.298024000 -1.963309000

C 6.346081000 3.453372000 -1.373582000

H 4.365657000 3.516785000 1.401231000

H 4.407539000 0.670488000 -1.805072000

H 6.195755000 4.766093000 0.322918000

H 6.211481000 1.954536000 -2.913776000

C -7.229115000 4.914768000 0.700297000

H -8.049978000 4.963577000 -0.017739000

H -7.656187000 4.645911000 1.672304000

H -6.803436000 5.916470000 0.798064000

C 7.464425000 4.215507000 -2.040519000

H 8.398956000 3.645956000 -2.011151000

H 7.238060000 4.412515000 -3.091907000

H 7.643799000 5.173192000 -1.548446000

# [Cd(L^2^)_2_Cl_2_] S = 0 *ttt* isomer (R = CH_3_)

Cd 0.168212000 -0.612224000 0.165878000

Cl -0.103270000 -0.547752000 -2.286120000

N -0.929939000 -2.994030000 0.127350000

N -2.435487000 -0.641974000 0.313698000

N -3.311862000 0.307678000 0.235888000

N -4.495607000 -0.261038000 -0.079891000

C -0.191490000 -4.102645000 0.157437000

H 0.880335000 -3.956555000 0.234889000

C -0.740339000 -5.380355000 0.102122000

H -0.097872000 -6.251587000 0.123937000

C -2.124311000 -5.500126000 0.025711000

H -2.593923000 -6.476331000 -0.013220000

C -2.900707000 -4.348957000 0.012133000

H -3.981390000 -4.414567000 -0.023245000

C -2.263365000 -3.104352000 0.060518000

C -3.022505000 -1.847729000 0.056074000

C -4.355647000 -1.606010000 -0.200267000

H -5.170537000 -2.243612000 -0.492683000

Cl 0.815607000 -1.206838000 2.474345000

N 0.260232000 1.904584000 0.592755000

N 2.522354000 0.392921000 -0.061153000

N 3.688136000 -0.109314000 -0.293980000

N 4.590498000 0.887756000 -0.136916000

C -0.836316000 2.608075000 0.888248000

H -1.770712000 2.058579000 0.880985000

C -0.806976000 3.967169000 1.184517000

H -1.724225000 4.491388000 1.421373000

C 0.420894000 4.620524000 1.168396000

H 0.488960000 5.678859000 1.393059000

C 1.563464000 3.896824000 0.858576000

H 2.531135000 4.382463000 0.835403000

C 1.449644000 2.530813000 0.575769000

C 2.625647000 1.717517000 0.249442000

C 3.965143000 2.043835000 0.203322000

H 4.505072000 2.950389000 0.409345000

C -5.664164000 0.535712000 -0.262860000

C -6.898715000 0.075807000 0.185301000

C -5.557528000 1.773088000 -0.897863000

C -8.033902000 0.855998000 -0.017533000

C -6.698326000 2.543409000 -1.077379000

C -7.956115000 2.100206000 -0.647661000

H -6.972661000 -0.870654000 0.707369000

H -4.591012000 2.110743000 -1.247861000

H -8.993260000 0.492744000 0.334201000

H -6.611381000 3.504138000 -1.573892000

C 5.982619000 0.638416000 -0.314825000

C 6.813748000 1.631446000 -0.826040000

C 6.502015000 -0.609205000 0.030038000

C 8.173656000 1.375404000 -0.976315000

C 7.858484000 -0.850146000 -0.141213000

C 8.720166000 0.134021000 -0.641563000

H 6.404288000 2.588413000 -1.126411000

H 5.841051000 -1.369104000 0.424544000

H 8.815660000 2.153672000 -1.374399000

H 8.257414000 -1.821890000 0.129875000

C 10.188770000 -0.152615000 -0.835381000

H 10.761923000 0.767660000 -0.962735000

H 10.603321000 -0.694927000 0.018248000

H 10.352665000 -0.771119000 -1.724116000

C -9.184667000 2.949360000 -0.861962000

H -10.080343000 2.459250000 -0.476646000

H -9.088458000 3.916379000 -0.359234000

H -9.343306000 3.151438000 -1.925469000

# [Zn(L^2^)_2_Cl_2_] di-molecular model S = 0 *ttt* isomer (R = CH_3_)

Zn -3.876300000 0.045700000 -1.415000000

Cl -4.697000000 0.291600000 -3.639300000

N -2.378700000 1.603700000 -1.542700000

N -2.067700000 -1.073600000 -1.883700000

N -1.727100000 -2.310100000 -2.069200000

N -0.390500000 -2.315100000 -2.266700000

C -2.610700000 2.912700000 -1.412800000

H -3.638200000 3.197400000 -1.219300000

C -1.599300000 3.860100000 -1.512900000

H -1.832400000 4.912400000 -1.410300000

C -0.295900000 3.422800000 -1.738000000

H 0.520600000 4.132200000 -1.805200000

C -0.044900000 2.064200000 -1.871200000

H 0.960800000 1.686400000 -2.017800000

C -1.122600000 1.180500000 -1.782800000

C -0.976100000 -0.261900000 -1.952800000

C 0.117700000 -1.058800000 -2.204400000

H 1.172300000 -0.832700000 -2.281900000

C 0.314700000 -3.535600000 -2.500500000

C 1.664200000 -3.624200000 -2.165400000

H 2.182700000 -2.784900000 -1.713500000

C 2.341500000 -4.814100000 -2.413400000

H 3.392100000 -4.879000000 -2.151800000

C 1.699700000 -5.919700000 -2.978100000

C 0.341800000 -5.800400000 -3.295300000

H -0.181300000 -6.642400000 -3.736600000

C -0.352900000 -4.619000000 -3.069300000

H -1.398400000 -4.526600000 -3.330000000

C 2.451700000 -7.196400000 -3.265200000

H 1.830600000 -8.075900000 -3.078400000

H 3.348700000 -7.277100000 -2.647700000

H 2.768800000 -7.237600000 -4.313000000

Cl -3.166800000 -0.233100000 1.157300000

N -5.281800000 -1.564800000 -0.945500000

N -5.608200000 1.098500000 -0.543700000

N -5.929000000 2.321000000 -0.276500000

N -7.148800000 2.287300000 0.297800000

C -5.059700000 -2.855700000 -1.200400000

H -4.104200000 -3.092700000 -1.653900000

C -5.990400000 -3.844900000 -0.901300000

H -5.771300000 -4.880700000 -1.127100000

C -7.196400000 -3.469300000 -0.317400000

H -7.946900000 -4.212000000 -0.072800000

C -7.430900000 -2.125900000 -0.054200000

H -8.362000000 -1.804700000 0.395700000

C -6.445400000 -1.194700000 -0.383000000

C -6.596100000 0.243100000 -0.156800000

C -7.601200000 1.008900000 0.389500000

H -8.568700000 0.769900000 0.792700000

C -7.795800000 3.492400000 0.705200000

C -8.521800000 3.524700000 1.891700000

H -8.566400000 2.650200000 2.529700000

C -9.161500000 4.702400000 2.268500000

H -9.723800000 4.723400000 3.195400000

C -9.074900000 5.857300000 1.487400000

C -8.325100000 5.798700000 0.305200000

H -8.242600000 6.681500000 -0.319900000

C -7.692500000 4.629700000 -0.095000000

H -7.125300000 4.585200000 -1.015300000

C -9.765900000 7.134600000 1.896300000

H -10.525400000 7.422400000 1.163000000

H -10.258400000 7.029300000 2.864400000

H -9.054800000 7.962600000 1.968300000

Zn 3.896600000 0.035800000 1.456000000

Cl 4.728700000 0.173800000 3.689300000

N 5.340100000 -1.528500000 0.947600000

N 5.601100000 1.151700000 0.607200000

N 5.875400000 2.383100000 0.329400000

N 7.098700000 2.391200000 -0.238800000

C 5.148100000 -2.830300000 1.170100000

H 4.183800000 -3.104500000 1.582200000

C 6.120000000 -3.785000000 0.891200000

H 5.924000000 -4.831100000 1.088500000

C 7.337400000 -3.361600000 0.366600000

H 8.120600000 -4.076300000 0.141700000

C 7.541000000 -2.007000000 0.138100000

H 8.480800000 -1.648500000 -0.263000000

C 6.513400000 -1.112700000 0.440100000

C 6.624500000 0.332000000 0.235700000

C 7.601000000 1.130600000 -0.315400000

H 8.551800000 0.918100000 -0.770000000

C 7.686000000 3.611900000 -0.687600000

C 9.047600000 3.836900000 -0.506700000

H 9.658100000 3.107000000 0.011400000

C 9.609500000 5.024800000 -0.964900000

H 10.669700000 5.198200000 -0.817100000

C 8.830700000 6.002500000 -1.589900000

C 7.460800000 5.755000000 -1.743700000

H 6.834100000 6.499400000 -2.223200000

C 6.884400000 4.569800000 -1.306300000

H 5.828200000 4.376100000 -1.438200000

C 9.445000000 7.282200000 -2.100700000

H 9.566400000 7.250200000 -3.188600000

H 10.430000000 7.454400000 -1.663100000

H 8.815400000 8.144900000 -1.869200000

Cl 3.176400000 -0.120400000 -1.112300000

N 2.389300000 1.570400000 1.694800000

N 2.101900000 -1.125600000 1.872100000

N 1.772700000 -2.373600000 1.987600000

N 0.436500000 -2.401800000 2.186600000

C 2.613900000 2.886800000 1.661100000

H 3.640700000 3.190600000 1.494200000

C 1.596700000 3.818000000 1.829700000

H 1.823500000 4.876500000 1.806100000

C 0.296300000 3.357500000 2.024500000

H -0.524400000 4.055000000 2.145100000

C 0.054400000 1.991100000 2.062600000

H -0.948100000 1.597900000 2.188700000

C 1.137200000 1.122700000 1.909300000

C 1.003000000 -0.328900000 1.988600000

C -0.083300000 -1.148600000 2.195500000

H -1.140100000 -0.937800000 2.286200000

C -0.256500000 -3.639300000 2.359100000

C -1.605600000 -3.724000000 2.020300000

H -2.132500000 -2.867900000 1.611100000

C -2.271600000 -4.930400000 2.211400000

H -3.321900000 -4.992100000 1.947600000

C -1.619400000 -6.056000000 2.722900000

C -0.262600000 -5.939800000 3.044700000

H 0.268500000 -6.797000000 3.445200000

C 0.421300000 -4.742400000 2.875000000

H 1.465900000 -4.653000000 3.140300000

C -2.361000000 -7.350400000 2.951400000

H -2.719200000 -7.419800000 3.984400000

H -3.231700000 -7.431100000 2.297000000

H -1.718800000 -8.215700000 2.770400000

1. [] Gaussian 09, Revision D.01, Frisch, M. J.; Trucks, G. W.; Schlegel, H. B.; Scuseria, G. E.; Robb, M. A.; Cheeseman, J. R.; Scalmani, G.; Barone, V.; Mennucci, B.; Petersson, G. A.; Nakatsuji, H.; Caricato, M.; Li, X.; Hratchian, H. P.; Izmaylov, A. F.; Bloino, J.; Zheng, G.; Sonnenberg, J. L.; Hada, M.; Ehara, M.; Toyota, K.; Fukuda, R.; Hasegawa, J.; Ishida, M.; Nakajima, T.; Honda, Y.; Kitao, O.; Nakai, H.; Vreven, T.; Montgomery, J. A., Jr.; Peralta, J. E.; Ogliaro, F.; Bearpark, M.; Heyd, J. J.; Brothers, E.; Kudin, K. N.; Staroverov, V. N.; Kobayashi, R.; Normand, J.; Raghavachari, K.; Rendell, A.; Burant, J. C.; Iyengar, S. S.; Tomasi, J.; Cossi, M.; Rega, N.; Millam, J. M.; Klene, M.; Knox, J. E.; Cross, J. B.; Bakken, V.; Adamo, C.; Jaramillo, J.; Gomperts, R.; Stratmann, R. E.; Yazyev, O.; Austin, A. J.; Cammi, R.; Pomelli, C.; Ochterski, J. W.; Martin, R. L.; Morokuma, K.; Zakrzewski, V. G.; Voth, G. A.; Salvador, P.; Dannenberg, J. J.; Dapprich, S.; Daniels, A. D.; Farkas, Ö.; Foresman, J. B.; Ortiz, J. V.; Cioslowski, J.; Fox, D. J. Gaussian, Inc., Wallingford CT, 2009. [↑](#endnote-ref-1)
2. [] F. Weigend, R. Ahlrichs, Balanced basis sets of split valence, triple zeta valence and quadruple zeta valence quality for H to Rn: Design and assessment of accuracy, Phys. Chem. Chem. Phys. **7** (2005) 3297-3305. [↑](#endnote-ref-2)
